# The immunomodulatory effect of oral NaHCO_3_ is mediated by the splenic nerve: multivariate impact revealed by artificial neural networks

**DOI:** 10.1186/s12974-024-03067-x

**Published:** 2024-03-28

**Authors:** Milena Rodriguez Alvarez, Hussam Alkaissi, Aja M. Rieger, Guillem R. Esber, Manuel E. Acosta, Stacy I. Stephenson, Allison V. Maurice, Laura Melissa Rodríguez Valencia, Christopher A. Roman, Juan Marcos Alarcon

**Affiliations:** 1 School of Graduate Studies & Department of Internal Medicine, Division of Rheumatology, SUNY Downstate Health Sciences University, Brooklyn, NY USA; 2 Division of Diabetes, Endocrinology, and Metabolic Diseases, NIH/NIDDK, Bethesda, MD USA; 3 Department of Medical Microbiology and Immunology, University of Alberta, Alberta, Canada; 4 Center for Studies in Behavioral Neurobiology, Concordia University, Montreal, Canada; 5 Mathematics and Computer Sciences Department, Barry University, Miami, FL USA; 6 Division of Comparative Medicine, SUNY Downstate Health Sciences University, Brooklyn, NY USA; 7 Department of Anesthesiology, Downstate Health Sciences University, Brooklyn, NY USA; 8 Department of Cell Biology, SUNY Downstate Health Sciences University, Brooklyn, NY USA; 9 Department of Rheumatology, SUNY Downstate Health Sciences University, 450 Clarkson Ave, Brooklyn, NY 11203 USA

**Keywords:** Inflammatory reflex (IR), Sodium bicarbonate (NaHCO_3_), Artificial neural networks, Splenic nerve, Spleen denervation, Cholinergic splenic anti-inflammatory pathway (CSAP), Splanchnic anti-inflammatory pathways (SAP), Vagal nerve stimulation (VNS)

## Abstract

Stimulation of the inflammatory reflex (IR) is a promising strategy for treating systemic inflammatory disorders. Recent studies suggest oral sodium bicarbonate (NaHCO_3_) as a potential activator of the IR, offering a safe and cost-effective treatment approach. However, the mechanisms underlying NaHCO_3_-induced anti-inflammatory effects remain unclear. We investigated whether oral NaHCO_3_’s immunomodulatory effects are mediated by the splenic nerve. Female rats received NaHCO_3_ or water (H_2_O) for four days, and splenic immune markers were assessed using flow cytometry. NaHCO_3_ led to a significant increase (p < 0.05, and/or partial eta squared > 0.06) in anti-inflammatory markers, including CD11bc + CD206 + (M2-like) macrophages, CD3 + CD4 + FoxP3 + cells (Tregs), and Tregs/M1-like ratio. Conversely, proinflammatory markers, such as CD11bc + CD38 + TNFα + (M1-like) macrophages, M1-like/M2-like ratio, and SSC^high^/SSC^low^ ratio of FSC^high^CD11bc + cells, decreased in the spleen following NaHCO_3_ administration. These effects were abolished in spleen-denervated rats, suggesting the necessity of the splenic nerve in mediating NaHCO_3_-induced immunomodulation. Artificial neural networks accurately classified NaHCO_3_ and H_2_O treatment in sham rats but failed in spleen-denervated rats, highlighting the splenic nerve's critical role. Additionally, spleen denervation independently influenced Tregs, M2-like macrophages, Tregs/M1-like ratio, and CD11bc + CD38 + cells, indicating distinct effects from both surgery and treatment. Principal component analysis (PCA) further supported the separate effects. Our findings suggest that the splenic nerve transmits oral NaHCO_3_-induced immunomodulatory changes to the spleen, emphasizing NaHCO_3_’s potential as an IR activator with therapeutic implications for a wide spectrum of systemic inflammatory conditions.

**Graphical abstract:**

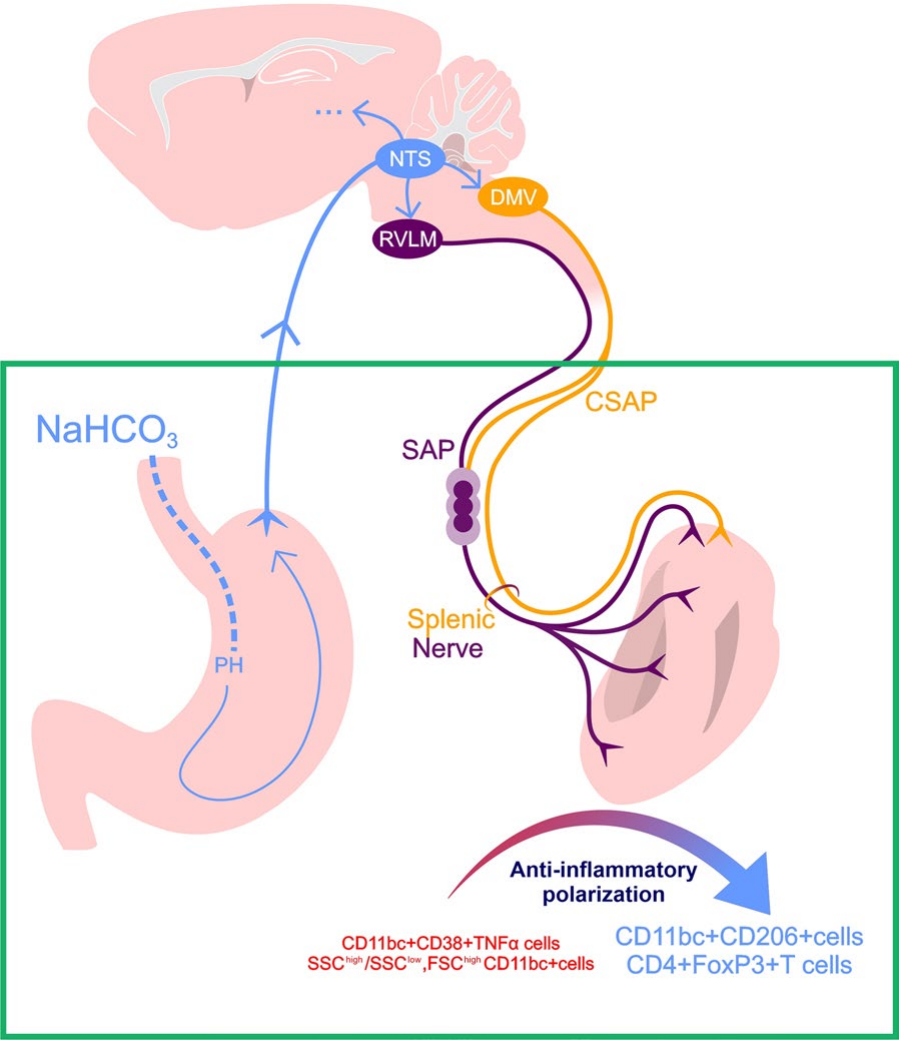

**Supplementary Information:**

The online version contains supplementary material available at 10.1186/s12974-024-03067-x.

## Highlights

- Oral sodium bicarbonate (NaHCO_3_) intake activates the inflammatory reflex.
- The activating mechanism remains unknown, limiting NaHCO3’s therapeutic application.
- First-time evidence of NaHCO_3_-anti-inflammatory effect is splenic nerve-mediated.
- Decreased granularity of CD11bc+FCShigh cells aligns with NaHCO_3_’s effect.
- Selective suppression of CD4+FoxP3+ and CD4-FoxP3+ T cells by spleen denervation

**Supplementary Information:**

The online version contains supplementary material available at 10.1186/s12974-024-03067-x.

## Introduction

A common denominator of systemic inflammatory conditions is an excessive systemic inflammatory response (SIR) [68, 78]. A promising new therapeutic field is SIR downregulation by stimulating the inflammatory reflex (IR), the intrinsic physiological neural mechanism that suppresses the production of proinflammatory cytokines [13, 16, 24, 25, 34, 57, 60–62, 70–72, 83, 84, 92]. The most widely explored IR activator is vagal nerve electrostimulation (VNS), which has been successfully used in experimental models of inflammatory disorders in animals and clinical trials in humans [7, 8, 10, 12, 14, 29, 30, 38, 40–42, 46, 56, 81, 87].

Recently, it has been suggested that oral NaHCO_3_ intake stimulates IR [65, 66]. This is highly significant because NaHCO3 is inexpensive, safe, and FDA-approved, ensuring a rapid translation into clinical practice. Oral administration of NaHCO_3_ solution in humans and rats induced an anti-inflammatory monocyte polarization relative to controls receiving the equivalent molar load of NaCl. Flow cytometry analysis in the spleen of NaHCO_3_-treated rats showed an increase in the total number of CD3 + CD4 + FoxP3 + lymphocytes (Tregs) and CD11b + CD206 + IL-10 + macrophages, whereas CD11b + F4/80 + TNF + macrophages decrease [65, 66].

The authors suggested that the immunomodulatory changes induced by oral NaHCO_3_ were IR-mediated through cholinergic splenic anti-inflammatory pathways (CSAP) [65, 66]. This was supported by a mitigated NaHCO_3_-mediated anti-inflammatory effect upon spleen displacement in association with the fibrosis of a layer of mesothelial cells attached to the spleen capsule. These cells stain positive for PGP9.5/choline esterase and lay over a dense network of nerves within the spleen capsule, thus, the authors hypothesized that the activation of mesothelial cells, by a yet unknown mechanism, releases acetylcholine near splenic nerve branches triggering CSAP. Nonetheless, spleen displacement may disrupt spleen innervation [23, 26]. Thus, it remains to be determined whether NaHCO_3_ can activate the IR and whether it does so via the activation of cholinergic mesothelial cells, splenic nerve branches, or both.

Here, we generated a sham (SH) and a spleen-denervated (SD) rat model that received four days ad libitum of either NaHCO_3_ or water (H_2_O). Flow cytometry analysis in the spleen replicated the NaHCO_3_-mediated immunomodulatory effect in SH but not in SD rats. Because both SD and SH models had disrupted connective tissue -hence mesothelial cells’ attachment to the spleen capsule- we argue for a necessary role of splenic nerve innervation to bring about NaHCO_3_’s effect. Furthermore, SD rats, independently from NaHCO_3_ treatment, modulate immunological markers.

Our study sheds light on the gut-brain-spleen communication responsible for NaHCO_3_’s effect on immune-cells-driven anti-inflammatory polarization. To the best of our knowledge, this is the first evidence that the immunomodulatory effect induced in the spleen by orally ingested NaHCO_3_ is mediated by the splenic nerve.

## Methods

### Rats

We used 8–12-weeks-old female (250–325 g, see Additional file 1: Fig. S1a) Sprague Dawley rats from Charles River. They were age-matched for all protocols and housed under standard conditions (12:12-h light–dark cycle and free access to food and water). All studies were conducted in accordance with the National Institutes of Health Guide for the Care and Use of Laboratory. All experiments were conducted under the Institutional Animal Care and Use Committee Animal Use Protocol (IACUC) #20-10574. This protocol was approved by the Office of Animal Welfare, Office of Research Administration from SUNY Downstate Health Sciences University.

### Surgeries and spleen collection

Rats underwent spleen denervation [26, 39, 69] confirmed by Western blotting for tyrosine hydroxylase (TH,anti-TH antibody; ab112; Abcam) as previously described [39, 85, 86]. For a description of the spleen denervation, sham surgeries, spleen collection, and immunoblotting see Additional file 1: Fig. S1a.

### Rat treatments

After sham (n = 24) and spleen denervation (n = 16) surgeries, animals were kept on LS chow with ab libitum water allowing 12–14 days of recovery. On the first day of the experiment, SH and SD rats were randomly assigned to drink a vehicle (H_2_O, n = 18) or 0.1 M NaHCO_3_ (n = 22) freshly made, ab libitum (see Additional file 1: Fig. S1a for daily consumption). This treatment was kept for 4 days, animals were euthanized on day 4 and spleens were harvested for flow cytometry analysis and western blot.

The concentration for the NaHCO_3_’s solution (equivalent to 8.4 mg of NaHCO_3_ in 1 L of water) is the highest dose used in rats by Ray et al. [65, 66]. By giving 0.1 M of NaHCO_3_ to rats we are below non-toxic levels in humans. A safe dose of NaHCO_3_ in chronic kidney disease (CKD) patients is 42 mg/kg/day which is ~ 3000 to 4000 mg of NaHCO_3_ daily [22], this amount is equivalent to 12.6 mg in rats. Our animals consumed ~ 0.42 mg of NaHCO_3_ per day which is 30 times less than 12.6 mg (non-toxic NaHCO_3_ levels in humans). This estimate is based on the amount of water consumed ad libitum by Sprague Dowley rats between 250 and 330 g of weight, which is 50 ml [51]. NaHCO_3_ has a molecular mass of 84.004 g/mol, and 50 ml of NaHCO_3_ at 0.1 M contains 0.42 mg of NaHCO_3_.

Ray et al. used NaCl at 0.1 M as a vehicle instead of H_2_O. We did not use NaCl to avoid the potential proinflammatory confounder effect of hypertonic sodium [33, 91].

### Flow cytometry

#### Tissue processing, antibodies, and technical information

We followed Ray et al. protocol with minor changes [65, 66]. Briefly, harvested spleens were processed and cell suspensions were incubated with antibodies and dead life solution to identify dead cells. For a full description of the flow cytometry protocol see Additional file 1: Fig. S1b, and for reagents and antibodies see Additional file 1: Fig. S2a.

To identify through flow cytometry macrophages residing in the spleen, we used antibodies against CD11bc, CD38, CD206, and TNFα. The CD38 and TNFα markers have been associated with a proinflammatory phenotype in monocytes and macrophages [31], whereas CD206 is associated with regulatory or anti-inflammatory properties [31]. The denomination of proinflammatory macrophages as M1 or anti-inflammatory as M2 oversimplifies the spectrum of phenotypes observed in these cells and there isn't a nomenclature agreed upon yet [49, 77]. For clarity, in the present work, we describe macrophages as M1-like (CD11bc + CD38 + TNFα +) and M2-like (CD11bc + CD206 +) as recently suggested [77].

Except for CD38, all the other markers have been used to identify NaHCO_3_-induced changes in rats [65, 66]. Ray et al. used anti-rat F4/80 antibodies for proinflammatory macrophages. At the moment of the current work, Novus had stopped the production of anti-rat F4/80 antibody and there was not a similar product available on the market. CD38 is considered selective for proinflammatory (LPS ± IFN-γ) but no anti-inflammatory macrophages (IL-4) [5], and transcriptomic analysis identified CD38 as a murine marker able to distinguish proinflammatory from anti-inflammatory-like macrophages [31]. To identify T cells, we used CD3, for T-helper we added CD4, and for Tregs we used FoxP3. We did not use CD8 but reported that CD4-T cells which include the CD8 + population.

#### Data acquisition

A Daily QC run was performed using Agilent Flow Cytometer QC particles to ensure the performance parameters meet the requirements. See Additional file 1: Fig. S2b for information about the flow cytometry setup. In flow experiments, the group identifiers were removed, and the analysis was performed by an investigator blind to the source of the samples. In each analysis, 500,000 total events were collected. Compensation beads (Thermo Fisher, Invitrogen™) were used to ensure that median fluorescence intensities of negative and positive were identical. We collected between 30,000 and 40000 events for compensation. Fluorescence Minus One (FMO) control was used to set the upper boundary for the background signal on the omitted label and to identify and gate the positive population. Samples were analyzed in duplicate measurements. Flow Cytometry was performed at the SUNY Downstate Health Sciences University, Flow Cytometry Facility, which received financial support from the Faculty of Medicine and grants from contributing investigators.

#### Data analysis

**Fig. 1:**
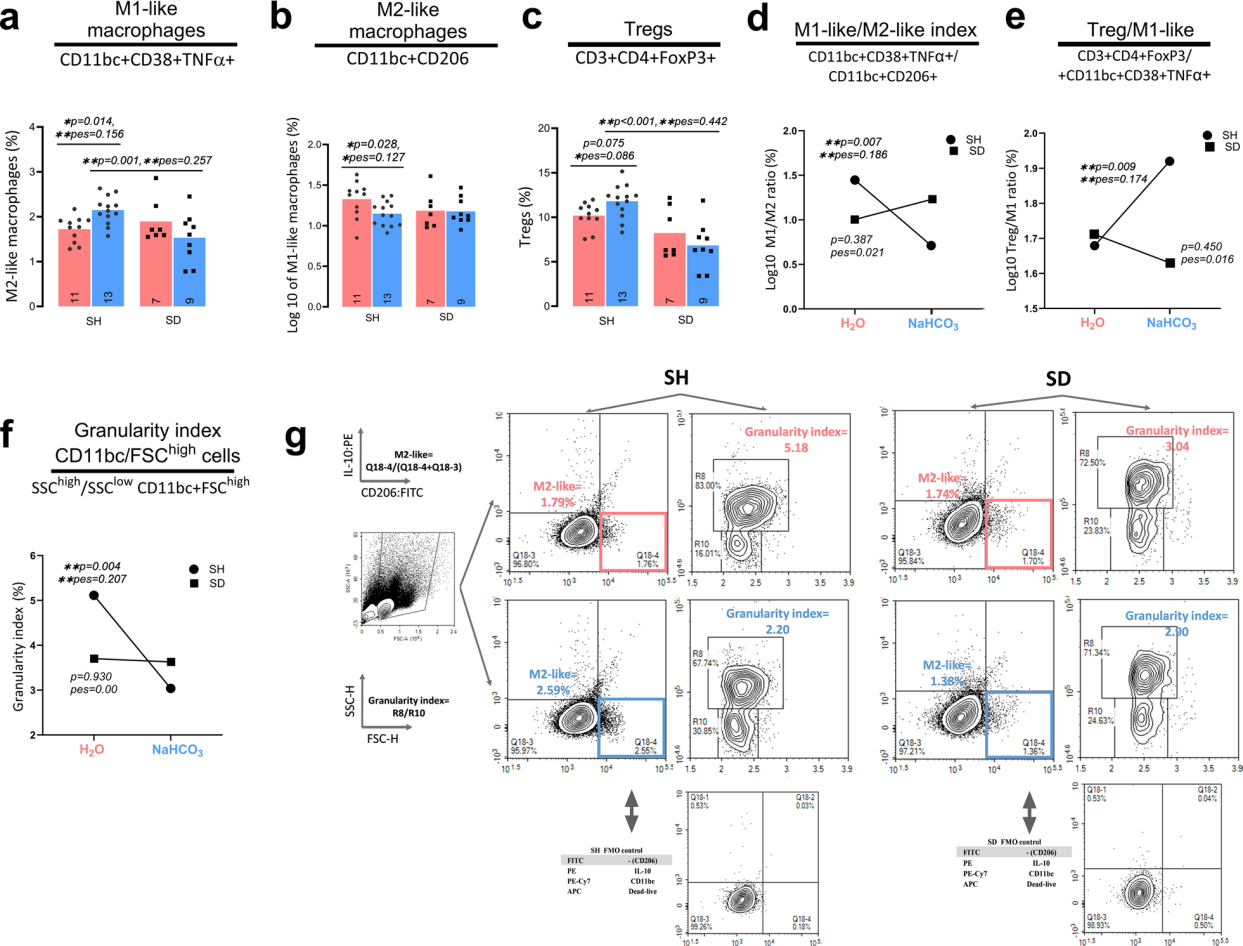
**The immunomodulatory effect induced by oral NaHCO**_**3**_ **on immune markers is abolished in SD animals. Spleen denervation has an independent immunomodulatory effect**. SD and SH rats were randomly assigned to drink H_2_O or NaHCO_3_, spleen was processed for immunophenotyping by flow cytometry. All values represent the percentage of live cells (an expanded gating for all cells can be seen in Additional file 1: Fig. S2a), with marker positivity listed below cell subtypes. The interaction was evaluated with a two-factor ANOVA (treatment; surgery). The interaction was considered present for p < 0.15 and pse > 0.04 and ANOVA was followed by a test of single effects (Table 1). Two-Factor ANOVA results: **a.** CD11bc + CD38 + TNFα + cells, interaction present, p = 0.137; **b** CD11bc + CD206 + cells, interaction present, p = 0.006**; **c** CD3 + CD4 + FoxP3 + cells, interaction present, p = 0.115; **d** M1-like/M2-like index, interaction present, p = 0.018*; **e** Log10 of the Treg/M1-like index, interaction present, *p = 0.023; **f** SSC^high^/SSC^low^ of CD11bc + FSC^high^ cells, interaction present, p = 0.070. The p and pse values of interest (2-factor ANOVA, and test of simple effects) are listed on each figure if respective group effects were found important (p < 0.05 and/or pse > 0.06). Percentages of M1-like and M2-like macrophages and granularity index are indicated in corresponding quadrants. **g** Representative gating for M2-like macrophages and granularity index in SH and SD rats, treated with H_2_O or NaHCO_3_. FMOs control for SH and SD for M2-like macrophages is shown. The colors light red and blue represent H_2_O and NaCHO_3_, respectively. At the left of the figure is the formula used to calculate macrophages and granularity index number and the y and x-axis labels. *Significant differences p < 0.05 or moderate SE (pse > 0.06); **p < 0.01 or large SE (pse > 0.14). Figure created with NovoExpress, and Prism GraphPad

**Fig. 2:**
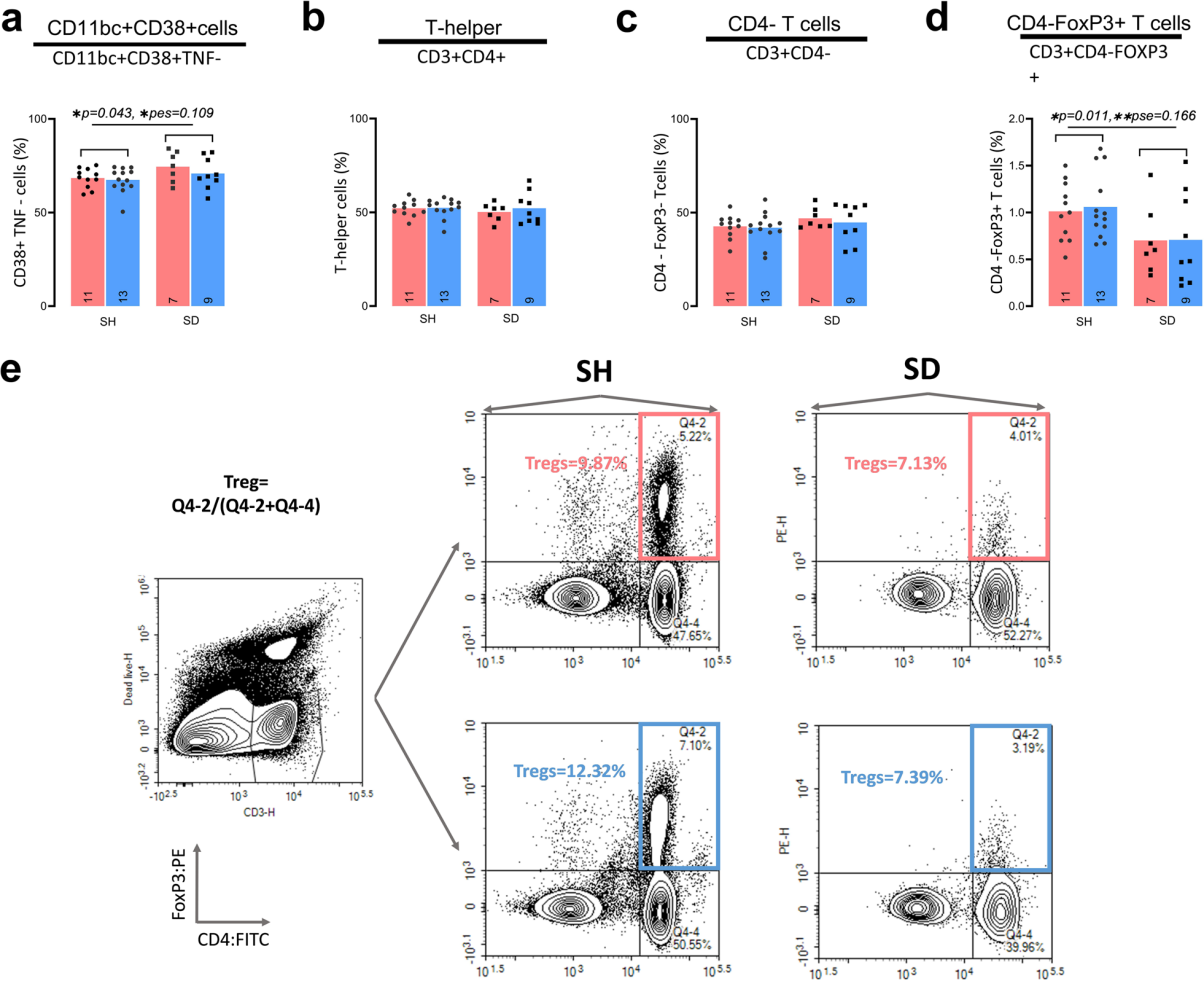
**The NaHCO**_**3**_ **treatment did not impact T-helper, CD11bc + CD38 + , CD4- T cells, and CD4-FoxP3 + T cells**. Spleen denervation increased CD11bc + CD38 + cells and suppressed CD4-FoxP3 + T cells. SD and SH rats were randomly assigned to drink H_2_O or NaHCO_3_, spleen was processed for immunophenotyping by flow cytometry. All values represent the percentage of live cells (an expanded gating for all cells can be seen in Additional file 1: Fig. S2a, b), with marker positivity listed below cell subtypes. The interaction was considered present for p < 0.15 and pse > 0.04. Two-Factor ANOVA results: **a** CD11bc + CD38 + cells, no interaction p = 0.559, treatment p = 0.317, surgery p = 0.043*; **b** CD3 + CD4 + cells, no interaction p = 0.690, treatment p = 0.632, surgery p = 0.509; **c** CD3 + CD4-cells, no interaction p = 0.777, treatment p = 0.564, surgery p = 0.168; **d** CD3 + CD4-FoxP3 + , no interaction p = 0.861, treatment p = 0.830, surgery *p = 0.011;. The p and pse values of interest (2-factor ANOVA, and test of simple effects) are listed on each figure if respective group effects were important (p < 0.05 and/or pse > 0.06). **e** Representative gating for Tregs isolated from the spleen of SH and SD rats, treated with H_2_O or NaHCO_3_. The color light red represents H_2_O and light blue NaCHO_3_. To the left is the formula used to calculate Treg’s final number and y and x-axis. *Significant differences p < 0.05 or moderate ES (pse > 0.06); ** p < 0.01 or large ES (pse > 0.14). Figure created with NovoExpress and Prism GraphPad

NovoExpress software was used to collect and analyze data. Dead cells and debris were excluded using forward and side scatterplots and dead life staining. Doublets were excluded with forward scatter height (FSC-H) and forward scatter area (FSC-A) plots. Representative gating images for M2-like macrophages and granularity index in SH and SD rats are shown in Fig. 1g while Tregs are shown in Fig. 2e. The granularity index in FSC^high^/CD11bc cells was determined after identifying two distinct cell populations based on granularity (SSC^high^/SSC^low^). After excluding debris and doublets we distinguish the alive populations (Additional file 1: Fig. S3a, gate R3) with lower FSC-A (y-axis) and higher FSC-A values (gate R6). We then assessed the degree of granularity (SSC values, Fig. 1B, y-axis) in both populations. Within R3 we gate agranular (SSC^low^) cells whereas R6 contained two populations, agranular and granular (SSC^high^). The cells inside R6 (R8 and R10 gates, Fig. 1g) are CD11bc positive and are the biggest. We identify that R8 contains those more granular cells. A summary of further gating of each cell is in Additional file 1: Fig. S3.

Cells expressing specific markers were reported as the ratio of the percentage of the events from the parent population in a specific quadrant or gate (positive population), divided by the same number plus the percentage of events from the parent population contained in the quadrant that did not express the marker (negative population). See Additional file 1: Fig. S2c for a detailed description of the proportion rate calculation for each cell type.

### Statistical analysis

Data for continuous variables are expressed as means ± standard deviation (SD). We used the Kolmogorov-Smirnov test to assess the normal distribution and we applied log10 transformation to those non-parametric variables (see Table 1). Logarithmic transformation was applied to variables with a non-parametric distribution resulting in a parametric distribution. A two-factor ANOVA was used to compare the four groups (SH surgery drinking H_2_O or NaHCO_3_, or SD drinking H_2_O or NaHCO_3_) and assess for interaction between treatment (H_2_O or NaHCO_3_) and surgery (SH, or SD). The test of single effects was used to discriminate the difference between groups if an interaction was present [32], Support, 2022 #306). One-way ANOVA was used to compare weight at euthanasia and amount of H_2_O or NaHCO_3_ solution consumption. The Levine test assessed for equal variances (see Table 1). Multivariate analysis was done using Artificial Neural Networks (ANN) and principal component analysis (PCA). We did not assess collinearity in the ANN models because of good predictive capacity in presence of collinearity [88]. We assessed multicollinearity before PCA excluding variables with a variance inflation factor (VIF) above 5 [88].

**Table 1:** Descriptive statistics, two-factor ANOVA, and test of simple effects analysis for immune markers after immunophenotyping with flow cytometry

	CD11bc + CD38 + cells	M1-like macrophage	M2-like macrophage	Granularity index in FSC^high^ cells	Macrophage polarization ratio	T-helper	Treg	CD4-T cells	CD4-FoxP3 + T cells	Treg/M1-like
	CD11bc + CD38 +	Log10 CD11bc + CD38 + TNFα +	CD11bc + CD206 +	CD11bc + SSC^high^/SSC^low^	Log10 M1-like/M2-like	Log10 CD3 + CD4 +	CD3 + CD4 + FoxP3 +	CD3 + CD4−FoxP3−	CD3 + CD4−FoxP3 +	Log10 Treg/M1-like
**Descriptive statistics**										
SH H_2_O (n = 11)	68.44 ± 5.29	1.33 ± 0.23	1.82 ± 0.74	5.22 ± 2.23	1.09 ± 0.27	2.72 ± 0.36	10.18 ± 1.44	42.61 ± 6.36	1.01 ± 0.31	1.67 ± 0.229
SH NaHCO_3_ (n = 13)	67.43 ± 6.62	1.15 ± 0.15	2.23 ± 0.43	2.87 ± 0.97	0.82 ± 0.17	2.72 ± 0.45	11.79 ± 1.86	41.85 ± 8.19	1.06 ± 0.35	1.92 ± 0.15
SD H_2_O (n = 7)	74.49 ± 8.16	1.18 ± 0.22	1.89 ± 0.49	3.70 ± 2.17	0.92 ± 0.26	2.68 ± 0.40	8.22 ± 2.84	46.94 ± 5.54	0.70 ± 0.37	1.71 ± 0.28
SD NaHCO_3_ (n = 9)	70.85 ± 8.33	1.18 ± 0.17	1.53 ± 0.55	3.63 ± 1.39	1.02 ± 0.25	2.71 ± 0.68	6.83 ± 2.61	44.72 ± 10.22	0.71 ± 0.49	1.63 ± 0.21
Levene’s Test, p	0.430	0.607	0.125	0.073	0.443	0.083	0.088	0.177	0.350	0.305
Distribution (KS), p	0.200	0.200	0.200	0.200	0.146	0.200	0.132	0.172	0.200	0.200
Two-factor ANOVA										
Interaction (Tr*Sx) p	0.559	0.137	0.005**	0.070	0.018*	0.690	0.038*	0.777	0.861	0.026*
pes	0.010	0.058	0.202**	0.088*	0.147**	0.004	0.115*	0.002	0.001	0.131*
(Tr) F	1.031					0.233		0.339	0.047	
p	0.317					0.632		0.564	0.830	
pes	0.028					0.006		0.009	0.001	
(Sx) F	4.400					0.446		1.980	7.160	
p	0.043*					0.509		0.168	0.011*	
pes	0.109*					0.012		0.052	0.166**	
Test of simple effects										
(Tr) SH H_2_OxNaHCO_3_, F		5.245	6.645	9.388	8.238		3.366			7.758
p		0.028*	0.014*	0.004**	0.007**		0.075			0.009**
pes		0.127*	0.156**	0.207**	0.186**		0.086*			0.174**
(Tr) SD H_2_OxNaHCO_3_, F		0.006	3.224	0.008	0.765		1.669			0.583
p		0.941	0.081	0.930	0.387		0.205			0.450
pes		0.000	0.082*	0.000	0.021		0.044			0.016
( Sx) H_2_O SHxSD F		2.384	0.798	3.124	2.491		3.565			0.095
p		0.131	0.378	0.086	0.123		0.067			0.760
pes		0.062 *	0.022	0.080*	0.065*		0.090*			0.003
(Sx) NaHCO_3_ SHxSD F		0.126	12.431	0.675	3.876		28.519			9.871
p		0.725	0.001**	0.417	0.057		< 0.001**			0.003**
pes		0.003	0.257**	0.018	0.097*		0.442**			0.215**

SH, animals with sham surgery; SD, animals with spleen denervation; Tr, treatment either H_2_O or NaHCO_3_; Sx, surgery either SD or SH; KS, Kolmogorov Smirnov; p, probability (significant for p < 0.05 except for the variables with an interaction between treatment and surgery where p < 0.15); pes, partial eta squared (small = pes < 0.06, medium = 0.06 < pes < 0.14, large = pes > 0.14); Tr, factor treatment which included either H_2_O or NaHCO_3_; Sx, factor surgery which included; *significant differences p < 0.05 or moderate size effect (pse > 0.06); **p < 0.01 or large SE (pse > 0.14)

To assess the magnitude of the difference between variables we used the effect size (ES) and the probability. The ES was measured by the partial eta squared (pes) and it was labeled “important” when pes was above 0.06 (moderate = 0.14 > pes > 0.06; high = pes > 0.14) [35]. The significance level was defined as p < 0.05. We provide both measurements in tables and Figures. All the graphs and data tables were designed using Prism GraphPad, and the statistical analysis was performed with SPSSv28 software.

## Results

### Effect of oral NaHCO_3_ intake in SH and SD animals

SD and SH rats were randomly assigned to drink a vehicle (H_2_O), or a solution of NaHCO_3_ (0.1 M) ad libitum for 4 continuous days. After spleen processing, immune cell phenotypes were identified by flow cytometry (see Additional file 1: Fig. S3b). Four groups of animals were stratified in 2 factors, surgery (SH or SD) and treatment (H_2_O or NaHCO_3_). The means and standard deviations for all variables are shown in Table 1. To control a possible interaction between spleen denervation and treatment response to oral NaHCO_3_, we used a two-factor ANOVA.

The variables with interaction (Table 1; p < 0.15, pes > 0.04) included CD11bc + CD38 + TNFα + cells (M1-like macrophages), CD11bc + CD206 + cells (M2-like macrophages), log10 of the M1/M2 ratio (M1-like/M2-like ratio), SSC^high^/SSC^low^ ratio in CD11bc + FSC^high^ cells (granularity index in CD11bc/FSC^high^ cells), CD3 + CD4 + FoxP3 + lymphocytes (Tregs), and the ratio of Tregs/M1-like macrophages (Tregs/M1).

Within this group, oral NaHCO_3_ decreased the fraction of M1-like macrophages and M1-like/M2-like ratio in SH animals compared to the H_2_O-treated group (Fig. 1a, d; stats), crucially, this effect was not observed in NaHCO_3_ and H_2_O treated-SD animals (Fig. 1s; stats). The granularity index of CD11bc/FSC^high^ also decreases in the NaHCO_3_-SH group (Fig. 1f; stats), while remaining unchanged in the SD animals (Fig. 1f; stats). This variable decreases in the same direction as proinflammatory monocytes, this is supported by a positive correlation with M1-like and negative with M2-like macrophages (Pearson coefficient 0.723, p < 0.01; − 0.522, p < 0.01 respectively) without collinearity with either macrophage subtype (Additional file 1: Fig. S5b). In addition, NaHCO_3_ treatment increased M2-like macrophages, Tregs, and Tregs/M1-like ratio in SH animals compared to the H_2_O group (Fig. 1b, c, e; stats); and like the M1-like findings, the NaHCO_3_-induced rise in M2-like, Treg, and Treg/M1-like was not observed in SD animals (Fig. 1b, c, e; stats).

The variables without interaction (Table 1; p > 0.15, pes < 0.04) between treatment and surgery involve CD11bc + CD38 + cells (CD11bc + CD38 + cells; Fig. 2A), CD3 + CD4 + lymphocytes (T-helper; Fig. 2B), CD3 + but CD4 negative lymphocytes (CD4- T cells; Fig. 2c), and lastly CD3 + FoxP3 + and CD4 negative lymphocytes (CD4-FoxP3 + T cells, Fig. 2d). None of these variables differed between the groups in response to treatment. Moreover, the T-helper and CD4-T cells were similar across the SH and SD animals for both H_2_O and NaHCO_3_ groups.

**Fig. 3:**
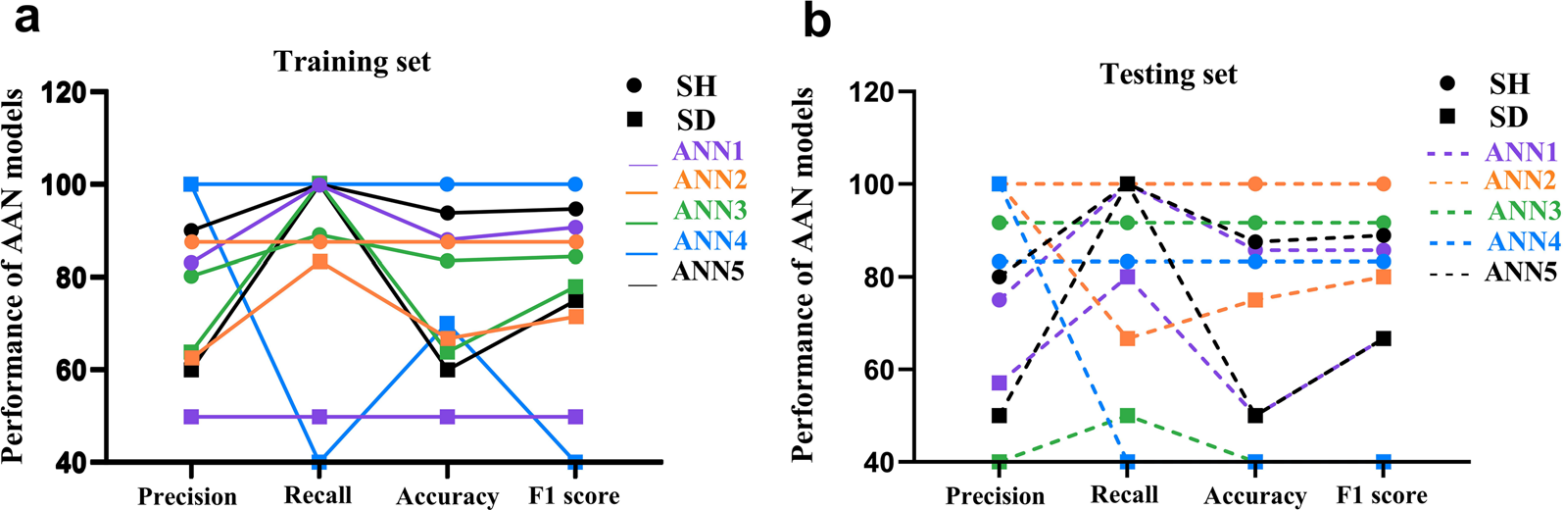
**Measurement tools for all the ANN models. a** Curves built with precision, recall, accuracy, and F1 score values for the training set are consistently above 80% for SH in comparison with the SD group. **b** Curves built with precision, recall, accuracy, and F1 score values for the testing set are consistently higher for the SH in comparison with the SD group. Figure created with Prism GraphPad

In summary, in the SH animals, treatment with oral NaHCO_3_ in comparison with H_2_O increased M2-like macrophages, Tregs, and Tregs/M1-like ratio while decreased M1-like macrophages, the granularity index of CD11bc/FSC^high^, and M1-like/M2-like ratio. Other cells were not affected including T-helper, CD11bc + CD38 + cells, CD4-T cells, and CD4-FoxP3 + T cells. In SD animals, the comparison of these immunological markers between H_2_O and NaHCO_3_ remained largely unchanged (Table 1, Figs. 2, and 3). Lastly, the interaction between surgery and treatment suggests an independent immunomodulatory effect induced by spleen denervation (Table 1).

### Effect of spleen denervation on immune markers.

This immunomodulatory effect of spleen denervation can be better seen by looking at the test of simple effects between SH and SD animals in each treatment group (Table 1). Within the H_2_O group, spleen denervation (compared to H_2_O/SH) moderately (0.14 > pes > 0.06) decreases M1-like macrophages, Tregs, and the granularity index of CD11bc/FSC^high^ cells, and did not change M2-like macrophages (Table 1). Within the NaHCO_3_ group, there were no changes (p > 0.05, and/or pes < 0.06) between SD and SH animals for M1-like macrophages or the granularity index. Contrary, SD rats have fewer M2-like macrophages and Tregs (p < 0.05, pes > 0.14, Table 1). These findings point to an effect of spleen denervation on immune markers in both H_2_O and NaHCO_3_-treated animals.

In the absence of an interaction between treatment and surgery (where the simple effect test is unnecessary), spleen denervation globally decreased CD4-FoxP3 + T cells and increased CD11bc + CD38 + cells in comparison to SH animals (Table 1). Other cells were unaffected by the surgery such as T-helper, and CD4-T cells. Overall, these analyses further indicate that spleen denervation, independently of the treatment effect (NaHCO_3_ or H_2_O), has an immunomodulatory effect.

### Identifying the multivariate effect of spleen denervation by artificial neural network (ANN) classification

Our findings are consistent with the immunomodulatory NaHCO_3_-mediated effects described by Ray et al. [65]. Crucially, our data shows the abolishment of this effect by spleen denervation. Next, we assess the same question with a multivariate approach. We assume that if the effect of NaHCO_3_ is mediated by the splenic nerve, a classificatory model integrated by multiple immune markers would generate accurate predictions in SH but not in SD animals. To build the model we used ANN [2, 27, 37, 43, 64]. We included those immune markers with an important difference (p < 0.05 and/or pes > 0.06) between NaHCO_3_ and H_2_O groups in the ANOVA results (Table 1).

The SH group has 11 and 13 animals subjected to H_2_O and NaHCO_3_ respectively, whereas the SD group has 7 and 9. The models were generated with SPSS running a simultaneous and parallel analysis for SH and SD groups. We ran five multilayer perceptron (MLP) ANN models (ANN1–ANN5) per group (Additional file 1: Fig. S4b, c). 70% of the sample was used to train the ANN models and 30% to test them which makes a rate of 2.3 (70/30 = 2.3). All models contained 6 independent variables, and the outcome-dependent variable was treatment (H_2_O or NaHCO_3_). To explore the overall performance of the ANN models (5 per group) we examined precision, recall, accuracy, and F1-score (HN, 2019).

All ANN models generated in SH performed consistently better than the models in SD animals (Fig. 3a, b). We selected ANN5 (ANN5-SH, and ANN5-SD) because it has the most comparable training and testing partition rates between SH and SD groups and is the closest to a 2.3 ratio (Additional file 1: Fig. S4a). Hence, a sample of 16 SH and 10 SD animals (66.7% and 62.5% respectively) randomly assigned by SPSS was used as the training, leaving aside 8 SH (33.3%) and 6 SD (37.5%) rats to validate ANN5-SH and ANN5-SD respectively (see network information in Additional file 1: Fig. S4b).

**Fig. 4:**
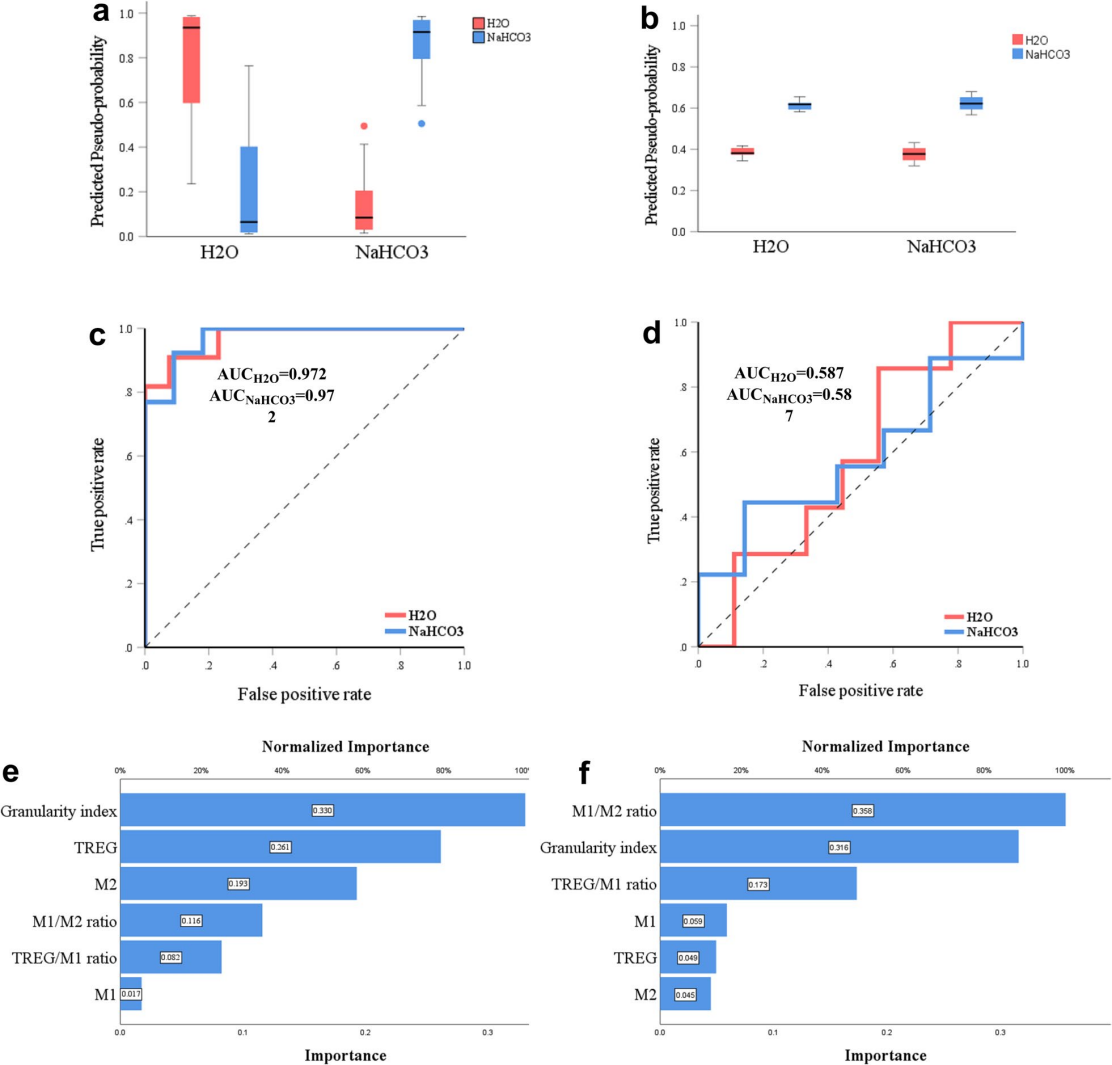
**Predictive pseudoprobability, ROC, and variables’ normalized importance for ANN5-SH and ANN5-SD models. a** The ANN5-SH’s predictive pseudo-probability to correctly classify rats drinking H_2_O or NaHCO_3_ is close to 100%, whereas the misdiagnosed cases were close to zero. **b** The ANN5-SD’s predictive pseudo-probability for correct predictions was close to 0.6 (60%) whereas the misdiagnosed cases were at 40%. **c** ANN5-SH’s ROC curve, representing the sensitivity (y-axis) and specificity (x-axis) for the model, is supported by an excellent AUC of 0.972. **d** ANN5-SD’s ROC curve and an unsatisfactory AUC of 0.587. **e, f** The importance of the 6 independent variables used to build the ANN5 models, **e** corresponds with ANN5-SH and **f** with ANN5-SD. Figure created with SPSS 28 and Prism GraphPad

Figure 4a, b display the predictive pseudo-probability for ANN5-SH and ANN5-SD. Within the H_2_O category, from left to right, the ANN5-SH model correctly classified more animals as drinking water (Fig. 4a; red boxplot in H_2_O category) than incorrectly classified them as drinking NaHCO_3_ (Fig. 4a; blue boxplot in H_2_O category). Consistently, within the NaHCO_3_ category, the ANN5-SH model correctly classified animals -with a predicted probability close to 1- as drinking NaHCO_3_ (Fig. 4a; blue boxplot in NaHCO_3_ category), and marginally incorrectly classified them -with a predicted probability close to zero- as drinking water (Fig. 4a; red boxplot in NaHCO_3_ category). In contrast, the ANN5-SD model showed a 0.6 probability of being classified as NaHCO_3_ and a 0.4 probability of being classified as H_2_O for either category, thus failing to correctly classify animals to each category (Fig. 4b).

The classificatory robustness of ANN5-SH and ANN5-SD was further compared by using the confusion matrix of cases classification, precision, recall, accuracy, F1 score, and area under the Receiver Operating Characteristics (ROC) curve (AUC), (HN, 2019). A case classification as a confusion matrix for both models is shown in Table 2. The outcome of the ANN models was defined as correct if the predicted probability was above 0.5. ANN5-SH correctly classified 15 out of 16 cases (93.8%) in the training and 7 out of 8 cases (87.5%) in the testing data sample (Table 2). In contrast, ANN5-SD correctly classified 6 out of 10 cases (60%) in the training set and 3 out of 6 cases (50%) in the testing set (Table 2). Thus, the ANN5-SH model performed substantially better than the ANN5-SD to classify treatment exposure, which is consistent with our prediction.

**Table 2:** Classification is reported as a confusion matrix

Set	SH				SD			
	Prediction				Prediction			
Actual	NaHCO_3_	H_2_O	Total	Correct (%)	NaHCO_3_	H_2_O	Total	Correct (%)
Training								
NaHCO_3_	9	0	9	100.0	6	0	6	100.0
H_2_O	1	6	7	85.7	4	0	4	0.0
Total	10	6	16		10	0	10	
Overall (%)	62.5	37.5		93.8	100.0	0.0		60.0
Testing								
NaHCO_3_	4	0	4	100.0	3	0	3	100.0
H_2_O	1	3	4	75.0	3	0	3	0.0
Total	5	3	8		6	0	6	
Overall (%)	62.5	37.5		87.5	100.0	0.0		50.0

The correct percentage of cases in the training set for the SH group is 93.8% in comparison with 60.0% in the SD group. For the testing set, the correct percentage predicted by the SH group is 87.5% in comparison with 50% for the SD group

Precision, recall, accuracy, and F1 score for ANN5-SH are above 90% in the training and above 80% in the testing set (Table 3). The F1 score, is above 90% in the training set and 88.9% in the testing set, which is considered very good and good respectively [74]. Contrary, the F1 score for ANN5-SD was lower at 75% and 66.7% (Table 3). Furthermore, ANN5-SH had an accuracy of 93.8% and 87.5% for ANN5-SH, over the 70% considered as good performance in machine learning [93]. In contrast, the accuracy for ANN5-SD was 60% for the training and 50% for the testing set (Table 3). The recall was 100% in the training and testing sets for both groups (Table 3).

**Table 3:** Precision, recall, accuracy, and F1 score for ANN-SH and ANN-SD animals’ models

	SH		SD	
	Training (%)	Testing (%)	Training (%)	Testing (%)
Precision	90.0	80.0	60.0	50.0
Recall	100.0	100.0	100.0	100.0
Accuracy	93.8	87.5	60.0	50.0
F1 score	94.7	88.9	75.0	66.7

The ANN5-SH and ANN5-SD models were further validated by examining the area under the Receiver Operating Characteristics (ROC) curve (AUC), which displays the relationship between true and false positive rates (HN, 2019). The prominent left-shift (from the diagonal representing equal true positive/false positive rate) of the ROC curves in the ANN5-SH model indicates high sensitivity and specificity, and more accurate classification for both the H_2_O and NaHCO_3_ categories (Fig. 3e). The AUC value, which estimates the quality of the classificatory model, for the ANN5-SH model was 0.972, which is considered excellent [9, 54]). In contrast, the ROC curves for either category in the ANN5-SD model remained unshifted (from the diagonal, Fig. 3f), and generated an AUC value of 0.587, which is unsatisfactory [9, 54].

Lastly, the importance of the 6 independent variables used to build the ANN5 models was ranked from high to low, those that contributed the most to ANN5-SH were the granularity index of CD11bc/FSC^high^ (100%), Tregs (79.1%), and M2-like macrophages (58.4%) (Fig. 4c). The variables that contributed most to the ANN5-SD model were the M1-like/M2-like ratio (100%), the granularity index of CD11bc/FSC^high^ (88.4%), and Tregs/M1-like ratio (48.5%) (Fig. 4d).

In summary, ANN models built on information from the SH group can correctly classify whether animals were drinking H_2_O or NaHCO_3_, but one built on the same information from the SD group failed to do so, which is consistent with a splenic nerve role in NaHCO_3_-mediated immunoregulation.

### Identifying immune markers clusters underlying immunoregulation by principal components analysis (PCA)

In “Effect of oral NaHCO3 intake in SH and SD animals” and "Effect of spleen denervation on immune markers" we identified interactions between treatment and surgery on several immune markers. We, therefore, wondered whether the immunoregulatory effects of spleen denervation and treatment are differentiated. We use PCA to address this question.

We first ran PCA with the 12 variables including 10 immune markers plus the type of treatment and surgery to examine the correlation matrix, collinearity, and anti-image matrices. Eleven of the 12 variables correlated above 0.3 (Additional file 1: Fig. S5a), 4 variables showed high M1-like multicollinearity coefficients (VIF > 5 and tolerance < 0.2; Additional file 1: Fig. S5b) and one variable (CD11bc + CD38 + cells) had an anti-image correlation matrix below 0.50 (Additional file 1: Fig. S5c). Based on this preliminary assessment, we excluded M1-like/M2-like ratio, Treg/M1-like ratio, T-helper, and CD4-T cells and CD11bc + CD38 + cells, keeping 7 variables for further analysis.

**Fig. 5:**
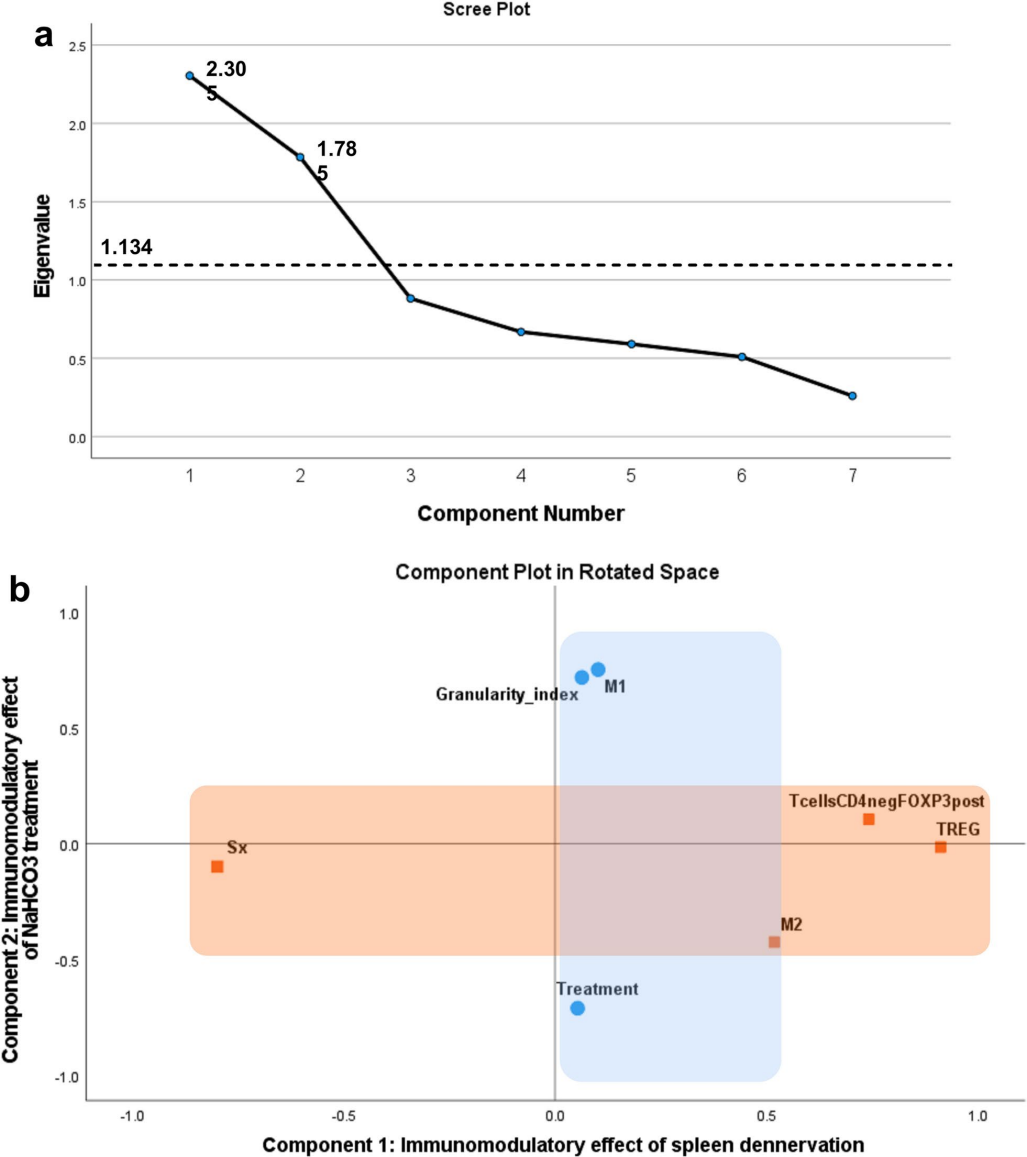
**Principal component analysis (PCA) for SH and SD groups together.** **a** Scree plot from PCA showing as many components as variables (x-axis), the parallel analysis for the eigenvalue (y-axis) for component 3 was 1.134 and a dashed line is at that level including only the first two components in the final PCA. **b** The components plot in rotated space including component 1 as an orange area (immunoregulatory effect of spleen denervation) and component 2 as a blue area (immunoregulatory effect of NaHCO_3_ treatment). The first component is loaded with Treg, CD4-FoxP3 + T cells, M2-like macrophages, and surgery (zero representing sham surgery and one spleen denervation). The second component is loaded with M1-like macrophages, the granularity index, and the treatment (zero representing H_2_O and one NaHCO_3_ treatment). The area where blue and orange overlap represents the only complex variable in the PCA, which is M2-like macrophages. These cells had a predominant loading factor of 0.518 on Component 1, however, the loading for Component 2 was close to 0.427. Figure created with SPSS 28, and Prism GraphPad

A new PCA with the 7 variables shows a Kaiser–Meyer–Olkin (KMO) measure of sampling adequacy of 0.64 (above the recommended value of 0.60) and a significant Bartlett’s test of sphericity (χ^2^ (21) = 59.607, *p* < 0.001). Screen plot and parallel test analysis [58, 59] identified two components with eigenvalues over 1.134 (Fig. 5a). The first and second components’ eigenvalues explained 33% and 25.5% of the variance respectively, producing a cumulative variance of 58.43% (Table 4). The commonalities are above 0.30 supporting that each variable shares common variance with others (Table 4).

**Table 4:** The loading factors, communalities, variance, and eigenvalues of PCA combining SH and SD animals in the same group

Variables	Component 1	Component 2	Communalities
PC-unrotated component matrix			
M1-like	0.107	0.748	0.572
M2-like	0.514	− 0.431	0.451
Granularity index	0.068	0.715	0.515
Treg	0.911	− 0.024	0.831
CD4-FoxP3 + T cells	0.742	0.097	0.560
Surgery	− 0.801	− 0.096	0.651
Treatment	0.047	− 0.713	0.511
PC-rotated component matrix^a^			
M1-like	0.101	**0.749**	
M2-like	**0.518**	− 0.427	
Granularity index	0.063	**0.715**	
Treg	**0.911**	− 0.017	
CD4-FoxP3 + Tcells	**0.741**	0.103	
Surgery	**− 0.800**	− 0.102	
Treatment	0.052	**− 0.713**	
Variance			
Proportion of variance (%)	32.932	25.497	
Cumulative variance (%)	32.932	58.429	
Eigenvalue (SD^2^)	2.305	1.785	

^a^Rotation converged in 3 iterations, rotation method quartimax with Kaiser Normalization; PC, principal component; SD, standard deviation; Factor loadings > 0.50 are bolded

An assessment of the components’ structure showed that an orthogonal rotation and quartimax offers the best-defined structure (Fig. 5b). The first component is loaded with Tregs, CD4-FoxP3 + T cells, and M2-like macrophages, in inverse correlation with the presence of spleen denervation (Table 4; orange rectangle in Fig. 5b). The second component is loaded with M1-like macrophages and the granularity index of CD11bc/FSC^high^ cells, both variables in negative correlation with the presence of NaHCO_3_ treatment (Table 4; blue rectangle in Fig. 5b). Notably, M2-like macrophages had a cross-loading correlation in both components (Table 4). The PCA’s composite reliability to assess internal consistency reliability [21, 79] is 0.837 and 0.769 for components one (4 items) and two (3 items), respectively, which are above 0.7 and considered adequate [1].

Because spleen denervation and NaHCO_3_ treatment modulate M2-like macrophages and Tregs, we conduct a PCA only in SH animals. We included the same variables used in the PCA analysis for both groups. For the SH group, the variable CD4-FoxP3 + T cells had an anti-image correlation matrix below 0.50 and was excluded. The KMO measure of sampling adequacy was 0.73, and Bartlett’s test of sphericity was significant (χ^2^ (10) = 43.750, *p* < 0.001). The parallel test identified a first component that explained 57.17% of the cumulative variance and had a composite reliability of 0.866 (Table 5). The second identified component minimally contributed to the cumulative variance (Eigenvalue cut-off of 1.233); thus, we retained only the first component. This unique component was loaded (> 0.45 loading factor) with the presence of NaHCO_3_ treatment, M2-like macrophages, and Treg, in inverse correlation with M1-like macrophages, and the granularity index (Table 5). In contrast, for the SD group, the KMO measure of sampling adequacy was 0.56 (below the recommended value of 0.60) and Bartlett’s test of sphericity was not significant (χ^2^ (10) = 6.960, *p* = 0.729), thus, we did not carry out further analysis.

**Table 5:** Loading factors, communalities, variance, and eigenvalues for PCA for the SH group alone

Variables	Component 1	Communalities
PC-unrotated component matrix		
M1-like	**− 0.792**	0.627
M2-like	**0.819**	0.671
Granularity index	**− 0.810**	0.656
Treg	**0.464**	0.215
Treatment	**0.830**	0.689
Variance		
Proportion of variance (%)	57.174	
Cumulative variance (%)	57.174	
Eigenvalue (SD^2^)	2.859	

Factor loadings > 0.45 are bolded

*PC* principal component, *SD* standard deviation

Overall, PCA analysis for all animals (SH and SD) treated with NaHCO_3_ or H_2_O distinguished two distinct clusters of factors underlying immunomodulation, and PCA analysis within the SH group produced a sole component. These analyses are supportive of a multivariate immunomodulatory effect of oral NaHCO_3_ treatment.

## Discussion

Ray et al. recently demonstrate a decrease in M1-like, and an increase in M2-like macrophages, and Tregs, in rats receiving 0.1-M NaHCO_3_ solution for three consecutive days [65]. The authors hypothesize IR activation as the possible mechanism responsible for this effect. Herein, we replicated the NaHCO_3_-mediated immunomodulatory effect described by Ray et al. in SH but not in SD animals. Thus, suggesting the necessity of the splenic nerve to bring about NaHCO_3_’s effect. Furthermore, SD, independently from NaHCO_3_ treatment, modulated some immune cells whereas others were unaffected. Taken together, these data shed light on the gut-brain-spleen communication responsible for NaHCO_3_’s effect on immune-cells-driven anti-inflammatory polarization. In addition to describing a sole immunomodulatory-spleen denervation effect on specific immune cells. To the best of our knowledge, this is the first evidence that the immunomodulatory effect induced in the spleen by orally ingested NaHCO_3_, is mediated by the splenic nerve.

### Immunomodulatory effect of oral NaHCO_3_ intake in SH animals

Ray et al. hypothesize that the effect of NaHCO_3_ in macrophage polarization was by IR activation [65, 66]. In the classic CSAP model [83], upon IR stimulation, CSAP converging into the splenic nerve releases norepinephrine (NE) which activates a subset of T cells in the spleen through β2-adrenergic receptors (β2AR). These T-cells release acetylcholine (ACh) which interact with α7 nicotinic acetylcholine receptors (α7nAChR) in spleen macrophages downregulating the production of proinflammatory cytokines but without increasing IL-10 production [70]. The IR can also be stimulated by splanchnic sympathetic pathways (SAP) which also feed the splenic nerve, increasing IL-10 production [48]. In parallel, NE can also bind to β2AR on macrophage s to induce an anti-inflammatory polarization [26], as well as to β2AR on Tregs, proliferation, and enhancing Treg function [19]. Furthermore, the activation of β2-receptors on splenic regulatory lymphocytes via splenic nerve-norepinephrine released upon vagal nerve stimulation suppresses LPS-induced inflammation, providing additional evidence for the anti-inflammatory properties of these cells [89].

In our study, oral NaHCO_3_ modulates M1-like macrophages, M2-like macrophages, Tregs_s,_ the ratios of M1-like/M2-like, Tregs/M1-like, and the granularity of CD11bc/FSC^high^ cells. Other cells were not affected including T-helper, CD11bc + CD38 + cells, CD4-T cells, and CD4-FoxP3 + T cells. These results suggest that NaHCO_3_ activates the IR.

Notably, we find that among all immunological variables quantified, the largest changes between H_2_O and NaHCO_3_ groups are observed in Tregs/M1-like and M1-like/M2-like ratios. We anticipate these results based on the single response of M1-like macrophages (suppression), M2-like macrophages (increase), and Tregs (increase) to oral NaHCO_3_ (Table 1, Figs. 1, 2). However, the expression of these immune markers as a ratio displays a bigger effect size (pes = 0.186, pes = 0.174,) and significance (p = 0.007, p = 0.009) between the treatment groups. Similarly, Ray et al. showed that the monocyte polarization was clearest when expressed as the number of M1-like/M2-like [65, 66]. In the same line, we surmise that the more important difference of Tregs/M1-like and M1-like/M2-like ratios in comparison with M1-like or Treg alone speaks of a functional link between monocytes and Tregs. Macrophages with a more anti-inflammatory phenotype and Tregs seem to regulate each other and generate an immunosuppressive loop through cytokines or cell-to-cell interaction [17, 45, 67, 75, 80, 82].

We found a novel decrease in the granularity index of CD11bc + /FSC^high^ cells in SH animals treated with NaHCO_3_ in comparison with the H_2_O group. We distinguished two cell populations based on granularity: agranular (SSC^low^) and granular (SSC^high^) and identified CD11bc-positive cells in the latter population, which were the biggest. Previously, a higher proportion of granular than agranular cells has been reported in tumoral cells in an inflammatory environment [47]. The granularity index correlates positively with M1-like and negatively with M2-like macrophages but without collinearity with either macrophage subtype. This suggests that changes in the granularity index of CD11bc + /FSC^high^ cells move in the same direction as M1-like cells but relying on a different type of cell (lack of collinearity), a potential suggestion is neutrophils. Consistent with this, Ray et al. described a significant decrease in neutrophils (CD16 + TNFα +) in the blood of human subjects after a load of oral NaHCO_3_ [65].

In addition to finding a sole effect under oral NaHCO_3_ intake, where the number of M2-like macrophages and Tregs increase while M1-like and the granularity index of CD11bc + /FSC^high^ cells decreases, we also find a multivariate association among these variables with treatment. In SH animals, PCA clusters in one component NaHCO_3_ treatment, Tregs, and M2-like macrophages in negative correlation with M1-like macrophages and granularity index of CD11bc + /FSC^high^ cells. We name this component immunomodulatory effect of NaHCO_3_ and suggests an orchestrating immunomodulatory role for NaHCO_3_ treatment. As discussed above, the increase in M2-like macrophages increments Tregs, which can further increase M2-like cells. This coordinating effect seems consistent with the notion that NaHCO_3_ triggers changes that match the effect of known IR activators [4, 76]. Accordingly, VNS, an established IR activator, reduces neutrophil migration [36], promotes microglial M2-like polarization [15], and reduces lung mRNA levels of M1-like macrophage markers, while increasing M2-like markers [44]. Furthermore, nicotine, another IR activator, reduces neutrophil recruitment during sepsis development [36], and induces both anti-inflammatory macrophage polarization [73] and Tregs number [52].

### Splenic nerve and oral NaHCO_3_ intake

NaHCO_3_-mediated immunomodulation of immunological markers bespeaks involvement of the IR pathway, but a critical test was to demonstrate the necessity of the splenic nerve to NaHCO_3_’s effect. SD animals that received NaHCO_3_ treatment did not show changes in M2-like macrophages, M1-like macrophages, granularity index of CD11bc + /FSC^high^ cells, M1-like/M2-like ratio, Tregs, or Tregs/M1-like ratio. Moreover, ANN analysis correctly classifies whether animals were drinking H_2_O or NaHCO_3_ only for the SH group but fails to do so for the SD group. As above, a PCA in the SH group gathers M1-like macrophages, granularity index of CD11bc + /FSC^high^ cells, M2-like macrophages, Tregs_,_ and NaHCO_3_-treatment in one component. However, an attempt to run PCA analysis in SD animals using the same variables fails.

These results demonstrate that the splenic nerve is necessary to observe oral NaHCO_3_-mediated immunomodulatory changes in the spleen. This effect seems independent from the mesothelial cells’ choline esterase + described by Ray et al. [65, 66]. We think so because moving the spleen to the midline of the abdominal cavity was not sufficient to disrupt the immunomodulatory shift seen in SH animals. The splenic nerve is a necessary part of the IR to suppress systemic inflammation. This has been tested in the context of endotoxemia for CSAP and SAP, the efferent arms of the IR. Ballinas-Rosas et al. demonstrated how the splenic nerve was required for CSAP control of TNFα during endotoxemia [69]. Similarly, Martelli et al. report that splenic nerve activity is dependent on inputs from sympathetic splanchnic nerves during endotoxemia, while is absent when splanchnic nerves have been severed. Moreover, 80% suppression of TNFα is only present in those animals with intact splanchnic nerves [48]. By denervating the spleen, we have disrupted SAP and CSAP as they converge in the splenic nerve. Thus, this finding brings strong support to classify NaHCO_3_ as an IR activator.

We have previously suggested a mechanistic model whereby oral NaHCO_3_ intake stimulates the IR, in which the immunomodulatory effects of NaHCO_3_ might be mediated by choline esterase + mesothelial cells attached to the spleen capsule as well as splenic nerve branches [4]. By stimulating vagal afferents NaHCO_3_ oral intake could activate the nucleus of the tractus solitaries and other IR-implicated brain regions, the output of which could trigger the IR via sympathetic (SAP) and parasympathetic (CSAP) pathways converging in the splenic nerve. Additionally, via impacting afferent vagus, NaCHO3 might stimulate other vagal anti-inflammatory pathways that are not dependent on the spleen [11, 50]. Further experimental assessment within the brain and in animal models of LPS-induced endotoxemia and systemic inflammatory conditions, with disruption of SAP or CSAP, is crucial to validate and refine the characterization of NaHCO_3_'s properties as an inflammatory reflex (IR) activator.

Due to successful outcomes in prior studies with inflammatory reflex (IR) activators, NaHCO_3_ holds promise for treating various inflammatory conditions, including chronic kidney disease, atherosclerosis, hypertension, coronary artery disease, stroke, cancer, diabetes mellitus type 2 (DM2), obesity, Alzheimer's disease, autoimmune diseases, and psychiatric and neurological disorders[28], Matei, 2022 #618, Annoni, 2019 #619, Li, 2022 #616, Reijmen, 2018 #620, Sorski, 2023 #621, Dai, 2020 #622, Vargas-Caballero, 2022 #623, Ng, 2020 #624, Cimpianu, 2017 #625,[90]. Furthermore, NaHCO_3_, commonly used for heartburn and metabolic acidosis, has mild side effects [18] and its role as an IR activator suggests a straightforward translation to clinical use, leveraging its FDA approval and cost-effectiveness. We have recently published a review article that extensively addresses the potential clinical relevance of NaHCO_3_ for treating various inflammatory conditions [4].

### Spleen denervation has an independent immunomodulatory effect

We found that spleen denervation-independent of H_2_O or NaHCO_3_ treatment- decreased M1-like macrophages, granularity index in CD11bc/FSC^high^ cells, Tregs, M2-like macrophages, and CD4-FoxP3 + T cells whereas T-helper and CD4-T cells remained unaffected. The effect induced by SD on proinflammatory markers (M1-like, and granularity index CD11bc + /FSC^high^ cells) was more notable when comparing SH-H_2_O with SD-H_2_O groups, which was expected as these makers decrease with NaHCO_3_ treatment. Contrary, NaHCO_3_’s enhancing effect on anti-inflammatory immune markers (M2-like macrophages, and Tregs) sets up a more evident difference between SH and SD animals in the NaHCO_3_ group (Table 1). The immunomodulatory SD effect was further evidenced by a PCA component that grouped Tregs, CD4-FoxP3 + T cells, and M2-like macrophages inversely correlated with the presence of spleen denervation.

Consistent with a lower Treg percent in our SD group, an increase in Tregs observed in mice exposed to repeated social defeat stress (RSDS) was abolished in SD animals, which also showed decreased levels of IL-2, IL-17A, and IL-22 (cytokines specific from T cells) whereas T helper cells, IL-6, TNF-α, and IL-10 were unchanged [20]. We also found decreased CD4-FoxP3 + T cells with SD which includes CD8 + FoxP3 + T cells [53]. The function of CD8 + FoxP3 + T cells is not well characterized; however, suppressive properties of CD8 + FoxP3 + T cells in vitro and in vivo have been reported [55], also transcriptomic analysis in murine-induced CD8 + FoxP3 + T cells resembles CD4 + Tregs [3]. Thus, spleen denervation impacts specialized T cells (Tregs, CD4-FoxP3 + T-cells) whereas T-helper and CD4-T cells remain unchanged.

CD11bc + CD38 + cells were increased by spleen denervation. CD38 is present in multiple cell types but is most abundant in hematopoietic cells [63]. In the immune cells, CD38 is expressed in B cells, DC, NK cells, T cells, monocytes, macrophages, and neutrophils among others [63]. This molecule can act as a receptor for CD31 and as a NAD-depleting enzyme having an important role during inflammation [31, 63]. Thus, CD11bc + CD38 + cells in our study may mostly include monocytes, macrophages, DCs, and possibly some NK, B, and T cells, which total number increase by spleen denervation.

A study from Kooijman et al. showed that selective parasympathetic spleen denervation (by clearing connective tissue in the poles of the spleen) increased the count of splenic DC, B cells, and T cells, and gene expression of proinflammatory cytokines in the liver and peritoneal leukocytes in comparison with SH [39]. Additional sympathetic spleen denervation (by clearing of connective tissue in the hilum and splenic arteries) increased circulating IL-1β and IL-6 [39]. Our surgical spleen denervation combined the two procedures completed by Kooijman et al. We did not measure cytokines, but except for an increase in CD11bc + CD38 + cells (potentially representing monocytes, macrophages, and DCs), the effect in other immune markers suggests a predominant immunosuppressive effect of spleen denervation.

Our data support an independent immunomodulatory effect of SD: it decreased more specialized and polarized cell types such as Tregs, CD4-FoxP3 + T cells, M2-like macrophages; it increased CD11bc + CD38 + cells whereas CD4-T cells and T-helper cells were unaffected. More complete phenotypic panels are needed to better characterize the impact of spleen denervation on these cell populations.

### Limitations

Our study has implications but is not without limitations. We did not carry full immunophenotyping for some of the cells reported in the study such as the granularity index of CD11bc + /FSC^high^ cells, CD11bc + CD38 + cells, and CD4-FoxP3 + T cells which limits and makes the interpretation speculative. We worked with female rats as this sex is predominantly affected by autoimmune and some other inflammatory conditions [6], future work needs to expand our results to males. Also, the multivariate analysis specifically PCA would have benefited from a bigger sample size to conduct confirmatory factor analysis. Despite these limitations, this study contributes to supporting the role of NaHCO_3_ as an IR activator which opens a wide broad spectrum of therapeutic possibilities.

## Conclusions

We report that the splenic immunomodulatory changes induced by oral NaHCO_3_ in SH are abolished in SD animals. This immunomodulatory effect is multivariate, where immune markers are grouped in the same component but proinflammatory cells are polarized on one side, inversely correlating with anti-inflammatory markers and NaHCO_3_ treatment located on the opposite side. Thus, further supporting an orchestrating immunoregulatory effect of oral NaHCO_3_. To our knowledge, this is the first evidence that the splenic nerve plays a necessary role in communicating signals induced from an oral basic solution to the spleen. This evidence may be enough to classify oral NaHCO_3_ as an IR activator. We also report an impact of spleen denervation on splenic immune markers, independent from treatment. This effect was immunosuppressive on more specialized T cells whereas other less specialized ones did not change.

### Supplementary Information

**Additional file 1: Figure S1. Spleen denervation, and flow cytometry protocol. a. **Cartoon representative of the splenic artery anatomy branching from the celiac artery. The dashed lines show where the surgery was performed at the apical and arterial splenic nerve branches. At the right of the cartoon, there is a complete description of the spleen denervation procedure. Confirmation of spleen denervation was done by western blot measuring TH protein (SH and SD rats are shown). Sham animals revealed TH whereas SD rats show a decrease or absence of TH. **b. **Flow cytometry protocol details. **Figure S2. Flow cytometry reagents, setup, and percentage rate calculation per cell type.** **a.** List of reagents used for flow cytometry. **b.** Laser lines, emission filters, and fluorochromes were used from Agilent NovoCyte 3000 flow cytometer. Figure created with CorelDraw, Microsoft Office, and Prism GraphPad. **c. **The formulas used to calculate the percentage rate c per cell type after acquisition and gating.** Figure S3**. **Flow cytometry gating strategy for immune markers was included in the study.** **a.** Flow cytometry gating for spleen macrophages. The number in the lower left corner represents the order of the gates. 1) FSC/SSC gate excludes debris/small particles; 2) FSC-H/FSC-A excludes doublets; 3) gating for alive cells with lower FSC (R3), and higher FSC (R6); 4a) CD11bc+ and lower FSC cells (R17); 4b) CD11bc+ and lower FSC cells (R7); 5a) M1-like macrophages (CD11bc+CD38+TNFα+) and CD11bc+CD38+ cells; 5b)M2-like macrophages (CD11bc+CD206+); 5c) index of the proportion of CD11bc+ higher FSC more granular (SSC^high^) cells (gate R8), and the proportion of CD11bc+ higher FSC less granular (SSC^low^) cells (gate R10); 6a) FMO control for M1-like macrophages; 6b) FMO control for M2-like macrophages. **b.** Flow cytometry gating for spleen T cells. 1) Gate for alive CD3+ cells; 2) FSC/SSC gate to exclude debris/small particles; 3) FSC-H/FSC-A to exclude doublets; 4) representative of T-helper, CD4-T cells, Tregs, and CD4-FOXP3+ T cells. All the gates and quadrants include the gated percentage. Almost all gates/quadrants include the number of events except for CD11bc+/CD38+ cells, T helper, CD4-T cells, and the FMO controls. The formula used to calculate a definitive number using negative and positive populations is also included with an arrow pointing to the respective quadrant or gate. Figure created with NovoExpress and Prism GraphPad. **Figure S4. Case processing summary for all AANs and network information for AAN5 per group.** **A. **Case processing for the ANN models. The random partition for training and testing sets is reflected for each ANN model in SH and SD groups. The model with the most comparable training and setting partition between SH and SD groups is ANN5. **b.** Network information for ANN5-SH and ANN5-SD. **c.** Network architecture for ANN5-SH (left) and ANN5-SD (right). Figure created with SPSS 28, Microsoft PowerPoint, and Prism GraphPad. **Figure S5. Correlation matrix, anti-image matrices, and collinearity statistics.** **a. **Correlation matrix between variables involved in PCA. The 1-tailed significance for the correlation is also reflected. **b.** The collinearity statistics table shows the VIF values. Those variables with VIF >5 such as t-helper, t-cytotoxic, M1-like/M2-like index, and Treg/M1 index were excluded from the PCA analysis. **c.** Anti-image matrices table, the values on the diagonal of the anti-image correlation are all above 0.5 except CD11bc+CD38+cells which were excluded from PCA. Figure created with SPSSv28 and Prism GraphPad.

## Data Availability

This study did not generate new unique reagents. The materials used are available as Additional tables within the study. Detailed data supporting the findings of this study are available from the corresponding author [MRA] on request.
