# Supplementary material for: The immunomodulatory effect of oral NaHCO3 is mediated by the splenic nerve: multivariate impact revealed by artificial neural networks
Source: J Neuroinflammation. 2024 Mar 28;21:79. doi: 10.1186/s12974-024-03067-x (PMC10976719; doi:10.1186/s12974-024-03067-x)
Supplement: Supplementary file 1 — Additional file 1: Figure S1. Spleen denervation, and flow cytometry protocol. a. Cartoon representative of the splenic artery anatomy branching from the celiac artery. The dashed lines show where the surgery was performed at the apical and arterial splenic nerve branches. At the right of the cartoon, there is a complete description of the spleen denervation procedure. Confirmation of spleen denervation was done by western blot measuring TH protein (SH and SD rats are shown). Sham animals revealed TH whereas SD rats show a decrease or absence of TH. b. Flow cytometry protocol details. Figure S2. Flow cytometry reagents, setup, and percentage rate calculation per cell type. a. List of reagents used for flow cytometry. b. Laser lines, emission filters, and fluorochromes were used from Agilent NovoCyte 3000 flow cytometer. Figure created with CorelDraw, Microsoft Office, and Prism GraphPad. c. The formulas used to calculate the percentage rate c per cell type after acquisition and gating. Figure S3. Flow cytometry gating strategy for immune markers was included in the study. a. Flow cytometry gating for spleen macrophages. The number in the lower left corner represents the order of the gates. 1) FSC/SSC gate excludes debris/small particles; 2) FSC-H/FSC-A excludes doublets; 3) gating for alive cells with lower FSC (R3), and higher FSC (R6); 4a) CD11bc+ and lower FSC cells (R17); 4b) CD11bc+ and lower FSC cells (R7); 5a) M1-like macrophages (CD11bc+CD38+TNFα+) and CD11bc+CD38+ cells; 5b)M2-like macrophages (CD11bc+CD206+); 5c) index of the proportion of CD11bc+ higher FSC more granular (SSChigh) cells (gate R8), and the proportion of CD11bc+ higher FSC less granular (SSClow) cells (gate R10); 6a) FMO control for M1-like macrophages; 6b) FMO control for M2-like macrophages. b. Flow cytometry gating for spleen T cells. 1) Gate for alive CD3+ cells; 2) FSC/SSC gate to exclude debris/small particles; 3) FSC-H/FSC-A to exclude doublets; 4) representative of T-helper, CD4-T c [file 12974_2024_3067_MOESM1_ESM.docx]

Additional file 1


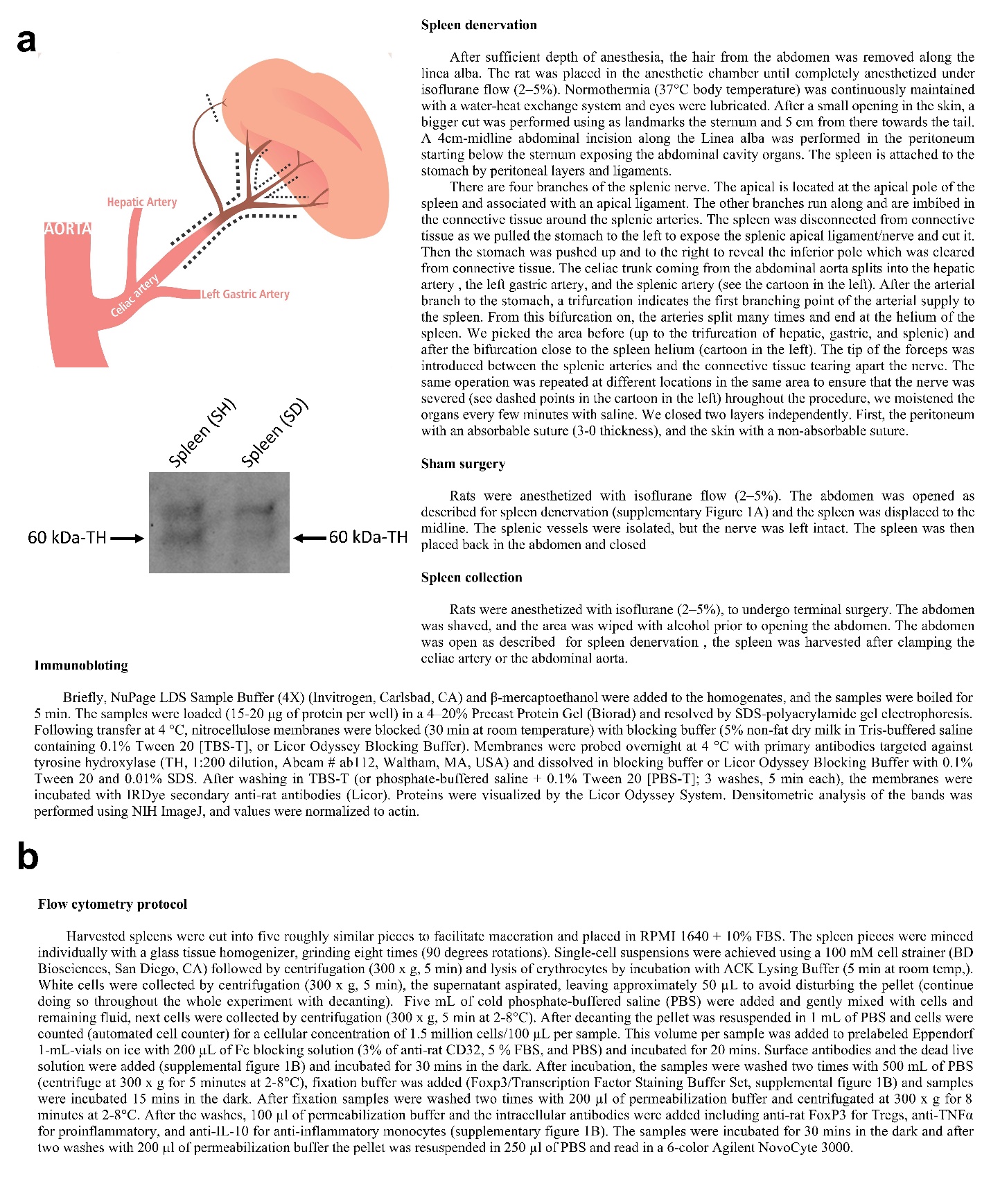
**Figure S1. Spleen denervation, and flow cytometry protocol. a.** Cartoon representative of the splenic artery anatomy branching from the celiac artery. The dashed lines show where the surgery was performed at the apical and arterial splenic nerve branches. At the right of the cartoon, there is a complete description of the spleen denervation procedure. Confirmation of spleen denervation was done by western blot measuring TH protein (SH and SD rats are shown). Sham animals revealed TH whereas SD rats show a decrease or absence of TH. **b.** Flow cytometry protocol details.


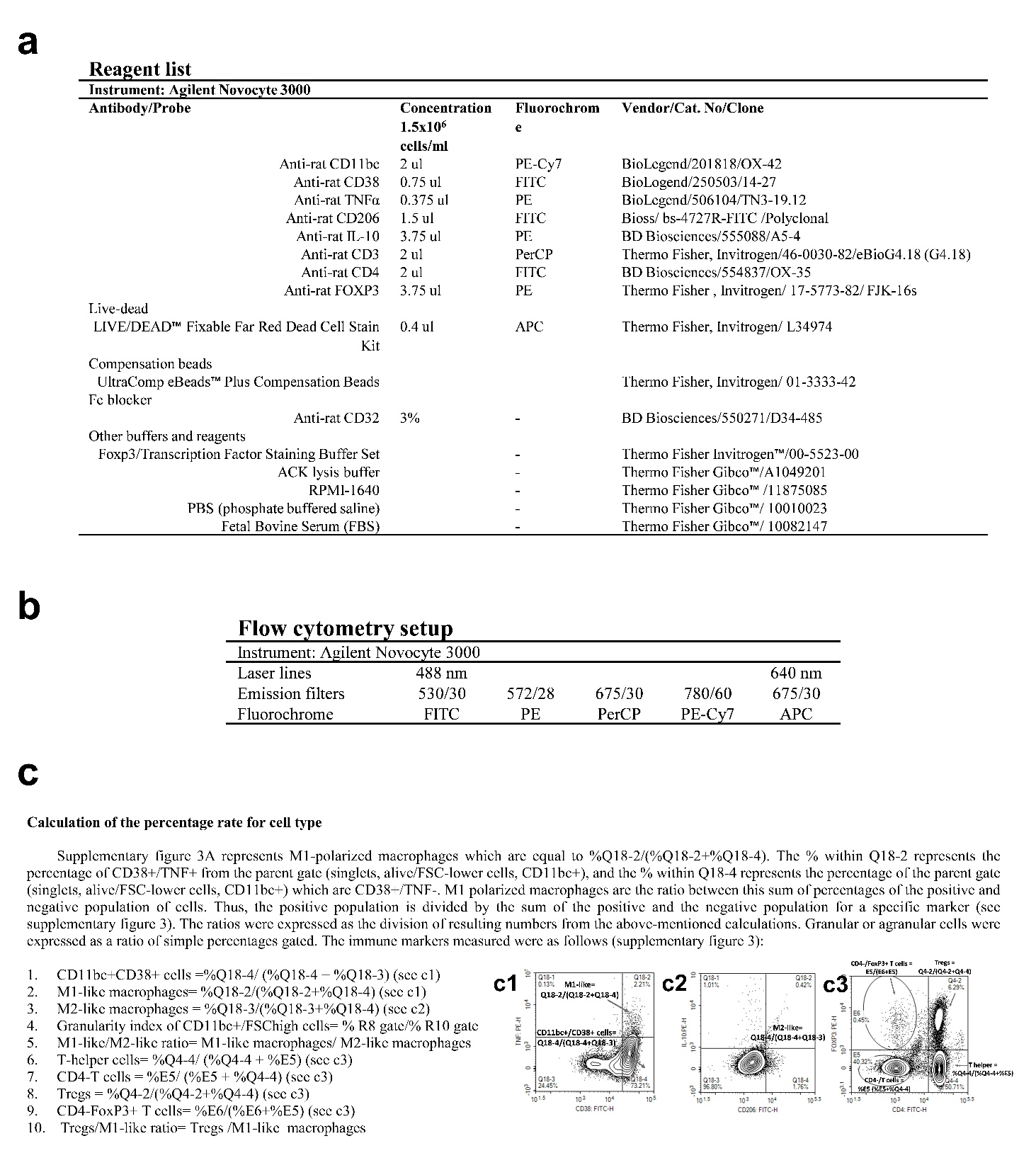
**Figure S2. Flow cytometry reagents, setup, and percentage rate calculation per cell type. a.** List of reagents used for flow cytometry. **b.** Laser lines, emission filters, and fluorochromes were used from Agilent NovoCyte 3000 flow cytometer. Figure created with CorelDraw, Microsoft Office, and Prism GraphPad. **c.** The formulas used to calculate the percentage rate c per cell type after acquisition and gating.

**
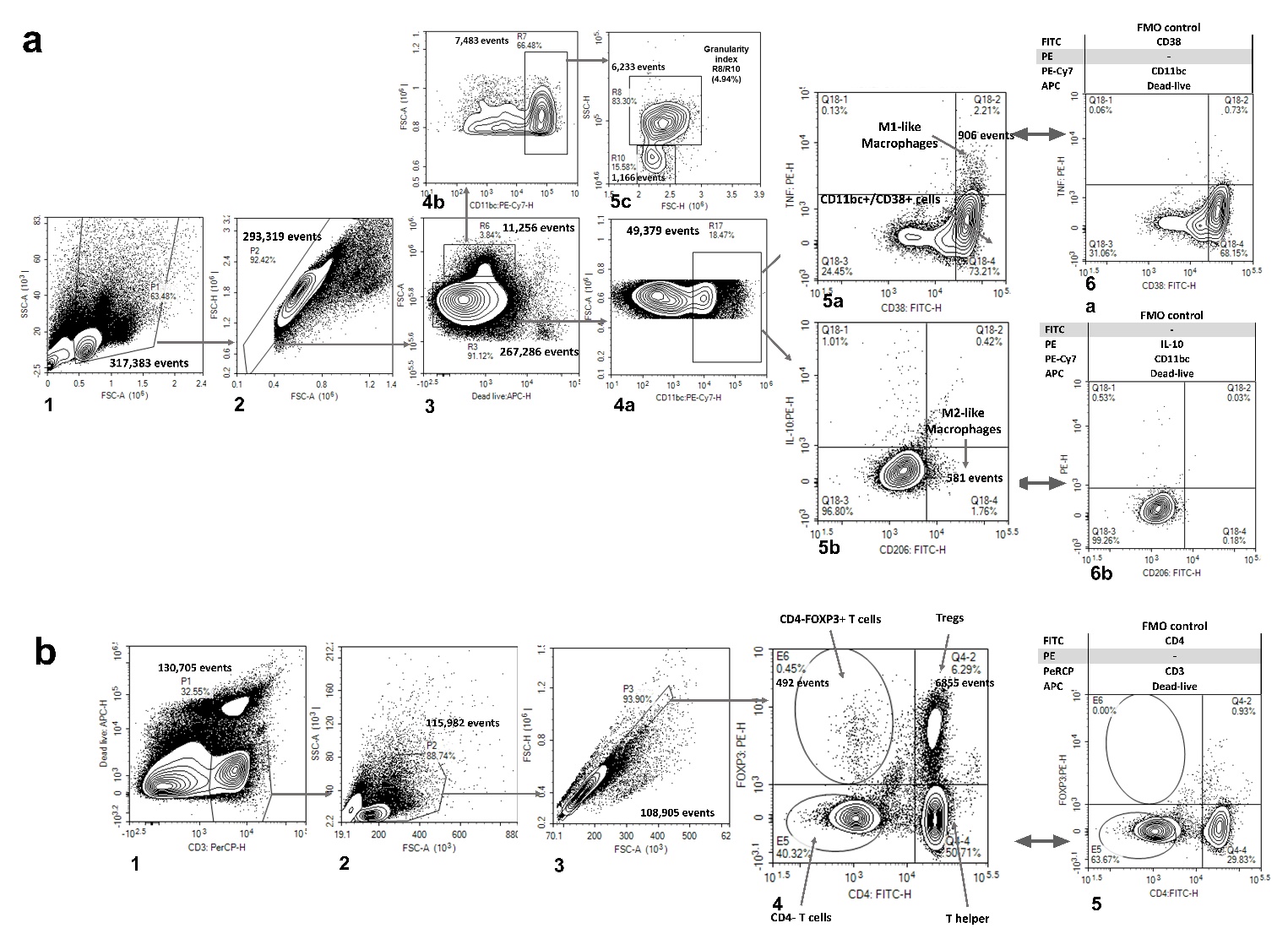
**

**Figure S3**. **Flow cytometry gating strategy for immune markers was included in the study.**

**a.** Flow cytometry gating for spleen macrophages. The number in the lower left corner represents the order of the gates. 1) FSC/SSC gate excludes debris/small particles; 2) FSC-H/FSC-A excludes doublets; 3) gating for alive cells with lower FSC (R3), and higher FSC (R6); 4a) CD11bc+ and lower FSC cells (R17); 4b) CD11bc+ and lower FSC cells (R7); 5a) M1-like macrophages (CD11bc+CD38+TNFα+) and CD11bc+CD38+ cells; 5b)M2-like macrophages (CD11bc+CD206+); 5c) index of the proportion of CD11bc+ higher FSC more granular (SSC^high^) cells (gate R8), and the proportion of CD11bc+ higher FSC less granular (SSC^low^) cells (gate R10); 6a) FMO control for M1-like macrophages; 6b) FMO control for M2-like macrophages.

**b.** Flow cytometry gating for spleen T cells. 1) Gate for alive CD3+ cells; 2) FSC/SSC gate to exclude debris/small particles; 3) FSC-H/FSC-A to exclude doublets; 4) representative of T-helper, CD4-T cells, Tregs, and CD4-FOXP3+ T cells. All the gates and quadrants include the gated percentage. Almost all gates/quadrants include the number of events except for CD11bc+/CD38+ cells, T helper, CD4-T cells, and the FMO controls. The formula used to calculate a definitive number using negative and positive populations is also included with an arrow pointing to the respective quadrant or gate. Figure created with NovoExpress and Prism GraphPad.


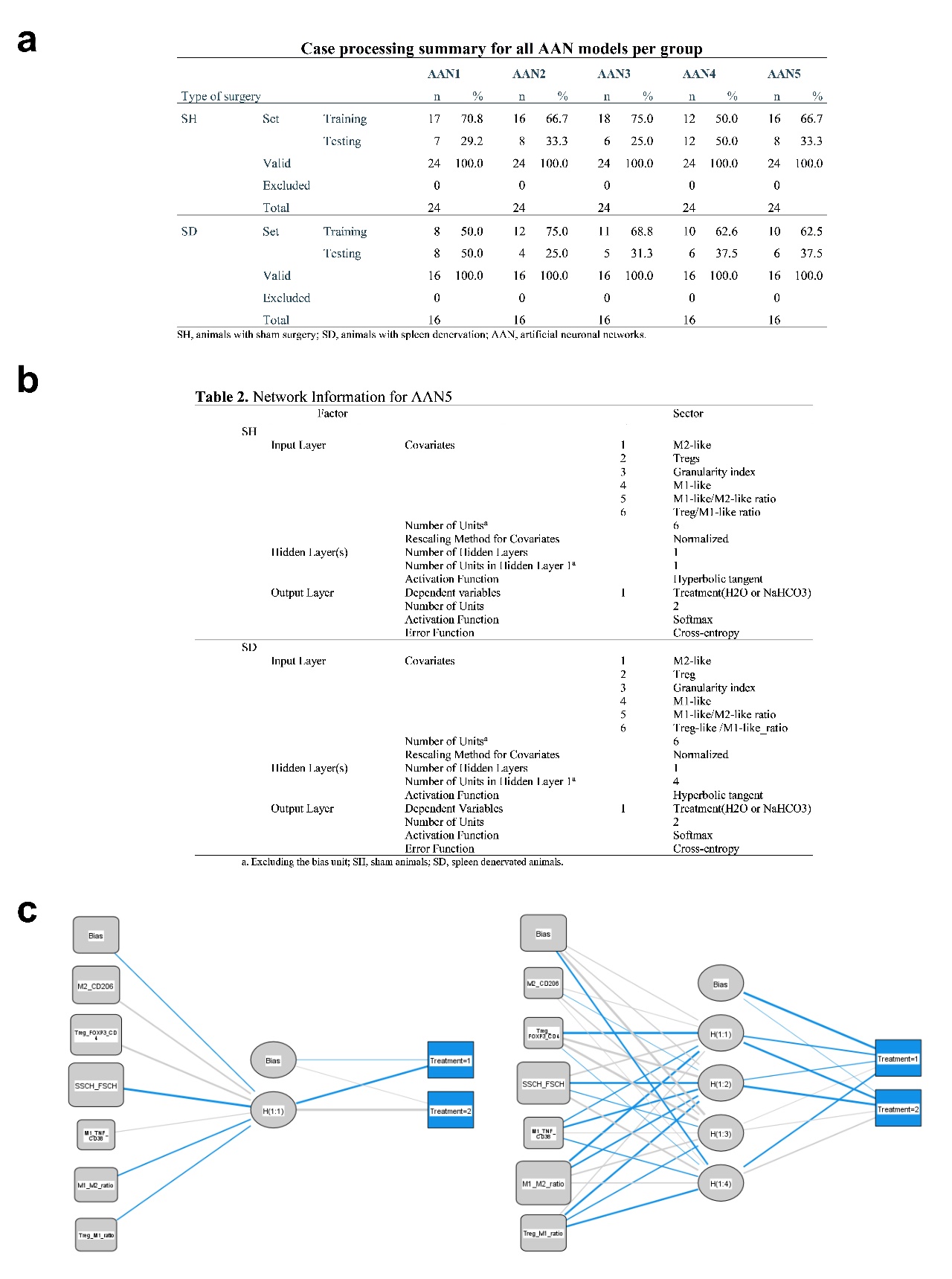


**Figure S4. Case processing summary for all AANs and network information for AAN5 per group. A.** Case processing for the ANN models. The random partition for training and testing sets is reflected for each ANN model in SH and SD groups. The model with the most comparable training and setting partition between SH and SD groups is ANN5. **b.** Network information for ANN5-SH and ANN5-SD. **c.** Network architecture for ANN5-SH (left) and ANN5-SD (right). Figure created with SPSS 28, Microsoft PowerPoint, and Prism GraphPad.

**
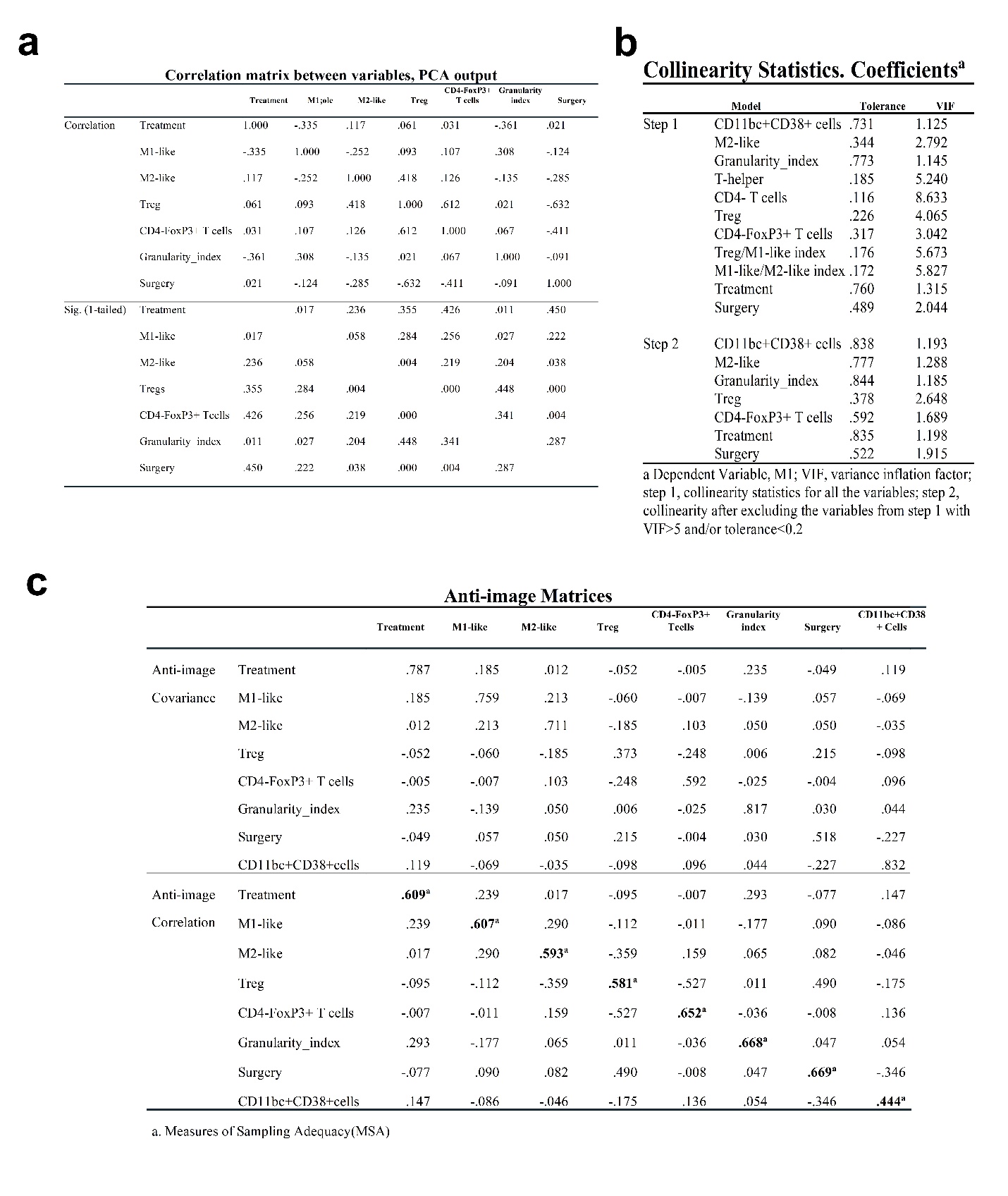
**

**Figure S5. Correlation matrix, anti-image matrices, and collinearity statistics. a.** Correlation matrix between variables involved in PCA. The 1-tailed significance for the correlation is also reflected. **b.** The collinearity statistics table shows the VIF values. Those variables with VIF >5 such as t-helper, t-cytotoxic, M1-like/M2-like index, and Treg/M1 index were excluded from the PCA analysis. **c.** Anti-image matrices table, the values on the diagonal of the anti-image correlation are all above 0.5 except CD11bc+CD38+cells which were excluded from PCA. Figure created with SPSSv28 and Prism GraphPad.
